# Structural Reconstruction of a Cobalt- and Ferrocene-Based Metal–Organic Framework during the Electrochemical Oxygen Evolution Reaction

**DOI:** 10.1021/acsami.4c03262

**Published:** 2024-07-23

**Authors:** Thomas Doughty, Andrea Zingl, Maximilian Wünschek, Christian M. Pichler, Matthew B. Watkins, Souvik Roy

**Affiliations:** † School of Chemistry, University of Lincoln, Green Lane, Lincoln LN6 7DL, U.K.; ‡ Institute of Applied Physics, TU Vienna, Wiedner Hauptstraße 8-10, Vienna 1040, Austria; § Centre of Electrochemical and Surface Technology, Viktor Kaplan Straße 2, Wiener Neustadt 2700, Austria; ∥ School of Mathematics and Physics, University of Lincoln, Lincoln LN6 7TS, United Kingdom

**Keywords:** coordination polymers, electrochemical restructuring, electrocatalysis, metal−organic frameworks, oxygen evolution reaction, X-ray absorption spectroscopy, SQUID

## Abstract

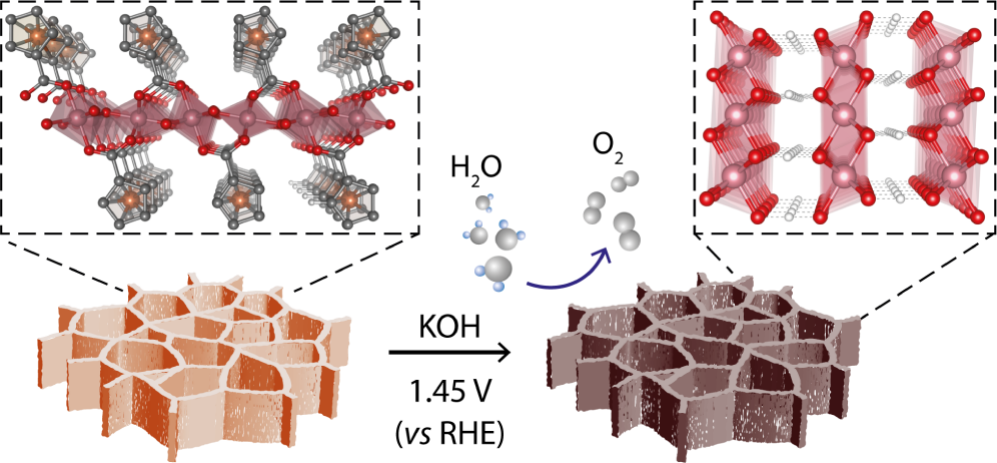

Metal–organic frameworks (MOFs) are increasingly being investigated as electrocatalysts for the oxygen evolution reaction (OER) due to their unique modular structures that present a hybrid between molecular and heterogeneous catalysts, featuring well-defined active sites. However, many fundamental questions remain open regarding the electrochemical stability of MOFs, structural reconstruction of coordination sites, and the role of *in situ-*formed species. Here, we report the structural transformation of a surface-grown MOF containing cobalt nodes and 1,1′-ferrocenedicarboxylic acid linkers (denoted as CoFc-MOF) during the OER in alkaline electrolyte. *Ex situ* and *in situ* investigations of CoFc-MOF film suggest that the MOF acts as a precatalyst and undergoes a two-step restructuring process under operating conditions to generate a metal oxyhydroxide phase. The MOF-derived metal oxyhydroxide catalyst, supported on nickel foam electrodes, displays high activity toward the OER with an overpotential of 190 mV at a current density of 10 mA cm^–2^. While this study demonstrates the necessity of investigating structural evolution of MOFs during electrocatalysis, it also shows the potential of using MOFs as precursors in catalyst design.

## Introduction

Electrochemical technologies are attracting considerable interest across the fields of chemistry, engineering, and energy, due to their importance in renewable energy conversion and storage in the context of greater supply of renewable electricity.^1^ Electrochemical water splitting, involving hydrogen evolution reaction (HER) at the cathode and oxygen evolution reaction (OER) at the anode, converts electricity into green H_2_, a storable chemical fuel and clean energy vector. The current water electrolyzer technology still has much room for improvement because the sluggish kinetics of the OER limit its performance and efficiency. Moreover, noble-metal-based catalysts (IrO_2_ and RuO_2_) are most commonly used in technologically mature electrolyzers, the cost of which affects their scalability.^2−4^ This has led to increased research effort toward developing low-cost, precious-metal-free catalysts for the OER as alternatives to IrO_2_ and RuO_2_.^5−7^ Successful implementation of water splitting technology also depends, to a large extent, on finding new electrocatalytic materials and robust electrodes with an open structure that will allow rapid release of bubbles and minimize catalyst spalling.^8,9^

The first-row transition-metal-based materials, such as metal oxides, hydroxides, chalcogenides, and phosphates, have attracted great attention, owing to their low cost and high electrocatalytic activities toward the OER.^10^ A key consideration in catalyst design is deciphering the catalytic motifs within the material that feature a range of surface functionalities and possible active sites. Organic/inorganic hybrid materials such as metal–organic frameworks (MOFs) and their derivatives are an emerging class of materials that are starting to find applications in electrochemical systems.^11−13^ MOFs, a type of coordination polymer with metal ions/clusters interconnected by organic ligands, offer an inherent advantage for catalyst design and performance optimization due to their unique structural versatility: their large surface area can expose abundant accessible sites for catalysis; their tailored pore structure and geometry can facilitate rapid transport of selected substrates and products throughout the bulk material; their molecular active sites at the nodes offer synthetic tunability and high atomic efficiency; and functionalization of the organic ligands can provide further structural selectivity to control reaction pathways. Furthermore, MOFs possess molecularly well-defined active sites and a tunable structure that would allow extraction of discrete structure–activity relationships. Taking OER as an example, a range of 3d transition-metal-based MOFs have been reported in recent years that display high electrocatalytic currents, and the coordinatively unsaturated metal nodes are commonly attributed to serve as the active sites.^14 −21^

However, with the increasing use of MOFs as electrocatalysts, questions have emerged regarding the structural robustness of these materials under operating conditions. The coordination bonds between the organic linkers and the metal nodes (or clusters) are generally more labile than ionic bonding in inorganic solids. Strongly alkaline conditions can promote the hydrolysis of the metal-linker coordination bonds, resulting in irreversible destruction of the framework structure. As recently suggested by many researchers, MOF particles can undergo *in situ* structural evolution to generate metal (oxy)hydroxide phases on the surface under harsh OER conditions.^22−28^ The as-formed metal (oxy)hydroxide is demonstrated to be the active catalyst for the OER, where the MOF scaffold not only acts as a precatalyst but also plays a critical role in regulating the formation of the nanostructured catalyst layer. Monometallic and bimetallic (oxy)hydroxide materials have been reported to be promising electrocatalysts for OER,^29^ but further electronic modulation and improvement of their performance via surface/interface engineering are challenging. Selection of MOFs as a sacrificial template to produce nanostructured metal (oxy)hydroxide under operating conditions thus presents a powerful strategy, because the intrinsic porosity and modular structure of MOFs might be able to tune the property of the evolved inorganic phases. However, deep insight into the role of intrinsic MOF structure in the morphological evolution process and its impact on the OER activity of the evolved catalyst is underexplored, and further understanding in this area can benefit next-generation catalyst design.

In this work, we have explored the structural transformation of a cobalt-based MOF (CoFc-MOF) containing 1,1′-ferrocene dicarboxylic acid (FcDA) linkers during OER. We were motivated by the recent research on 2D MOF nanosheets containing ferrocene-based organic linkers and nickel nodes that exhibit high activity and robust performance toward OER.^28,30^ Liang et al. proposed that Ni active sites engage in catalysis with electron-rich ferrocene linkers, facilitating the electron transport through the film.^30^ In contrast, Ding et al. demonstrated *in situ* formation of metal (oxy)hydroxide active catalyst from a defect-engineered nickel-ferrocene MOF.^28^ Herein, we present a comprehensive discussion of the morphological and structural evolution of CoFc-MOF during electrochemical treatments. In alkaline electrolyte (1 M KOH), CoFc-MOF grown on a nickel foam exhibits excellent catalytic activity toward OER with an overpotential of ∼190 mV at a current density of 10 mA cm^–2^, and the activity was maintained at ∼95% even after 24 h. The restructuring of the MOF to mixed metal oxyhydroxide was investigated by using a range of spectroscopic and analytical techniques, including X-ray absorption spectroscopy (XAS). A two-phase structural transition is observed at metal nodes, with Fe(III)-doped Co(OH)_2_ forming at the resting potential in alkaline electrolyte and Fe(III)-doped cobalt oxyhydroxide [Co(Fe)OOH] forming at oxidizing potentials. The *in situ*-formed Co(Fe)OOH exhibits oxygen vacancies in the lattice, promoting high OER activity.

## Experimental Section

### Materials

1,1′-Ferrocenedicarboxylic acid (FcDA) was synthesized following the literature procedure.^31^ FcDA was purified via an acid–base extraction.

Anhydrous cobalt(II) chloride (CoCl_2_, ≥98%) was purchased from Merck. Ethanol absolute (EtOH, ≥ 99.8%), acetone (≥99.8%), and hydrochloric acid (HCl, ∼37%) were purchased from Fisher Scientific. λ^2^-Cobalt(II) dihydroxide (97%) was purchased from Thermo Fisher Scientific. N,N-Dimethylformamide (DMF, Peptide grade) was purchased from Rathburn Chemicals. An Elga Purelab purification system was used for all deionized (DI) water (15 MΩ cm at 22 °C). Fluorine-doped tin oxide-coated glass (FTO) was purchased from Merck. Nickel foam (NF) was purchased from Nanographenex (>99.99%, 1.6 mm thickness, surface density 304 g m^–2^, ≥95% porosity). Carbon paper was purchased from the Fuel Cell Store (AvCarb P50T and AvCarb GDS2120).

### Synthesis of CoFc MOF

CoCl_2_ (35 mg, 0.27 mmol), FcDA (35 mg, 0.13 mmol), DMF (2 mL), and deionized water (1 mL) were mixed in a vial, and the vial was sealed with a screwcap lined with PTFE septa. The mixture was sonicated until the dissolution of the solids. The vial was placed in an oven, heated from room temperature (∼20 °C) to 120 °C at a ramp rate of 2 °C min^–1^ ramp rate, and held at 120 °C for 12 h. CoFc MOF (51 mg) was isolated as a dark brown powder via filtration and washing with DMF, deionized water, and EtOH.

### Electrode Preparation

NF was cut into 3 × 1 cm^2^ pieces and cleaned by soaking in 1 M HCl, acetone, DI water, and absolute ethanol, respectively, for 15 min in an ultrasonic bath. The cleaned NF was subsequently dried in a vacuum oven at 60 °C for 6 h and used the same day. FTO-coated glass slides were cut into 3 × 1 cm^2^ pieces and cleaned by heating at 70 °C in a 5:1:1 (v/v) solution of H_2_O:H_2_O_2_(30 wt %):NH_4_OH(conc.) for 30 min, followed by rinsing with DI water, ethanol, and acetone. The electrodes were dried under a N_2_ flow and stored at room temperature. To prepare MOF coated NF or FTO electrodes (denoted as NF|CoFc-MOF or FTO|CoFc-MOF), a clean NF or FTO electrode was placed into a vial containing MOF synthesis solution, which was subsequently heated in an oven at 120 °C for 12 h. For spectroelectrochemistry, the electrode was prepared by drop-casting 0.05 mL of catalyst ink on carbon paper (AvCarb P50T) over an area of 0.8 × 0.8 cm^2^. The ink for SEC was prepared by dispersing 2 mg of CoFc-MOF in 0.4 mL of IPA containing 0.005 mL of 5 wt % Nafion solution using 30 min sonication. Electrodes were allowed to dry at room temperature overnight. For X-ray absorption spectroscopy analysis of post-catalysis material, 0.25 mL of catalyst ink containing CoFc-MOF (16 mg of CoFc-MOF dispersed in 2 mL of IPA containing 0.04 mL of 5 wt % Nafion solution using 30 min sonication) was drop-cast on 1 × 1 cm^2^ carbon paper electrode (AvCarb GDS2120), which was subjected to 6 h controlled potential electrolysis at 1.5 V (*vs* RHE).

## Results and Discussion

### Synthesis and Characterization

**Figure 1:**
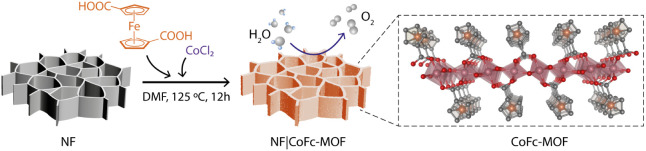
Illustration of the synthesis of CoFc-MOF on nickel foam (NF), which was utilized for electrochemical oxygen evolution reaction.

**Figure 2:**
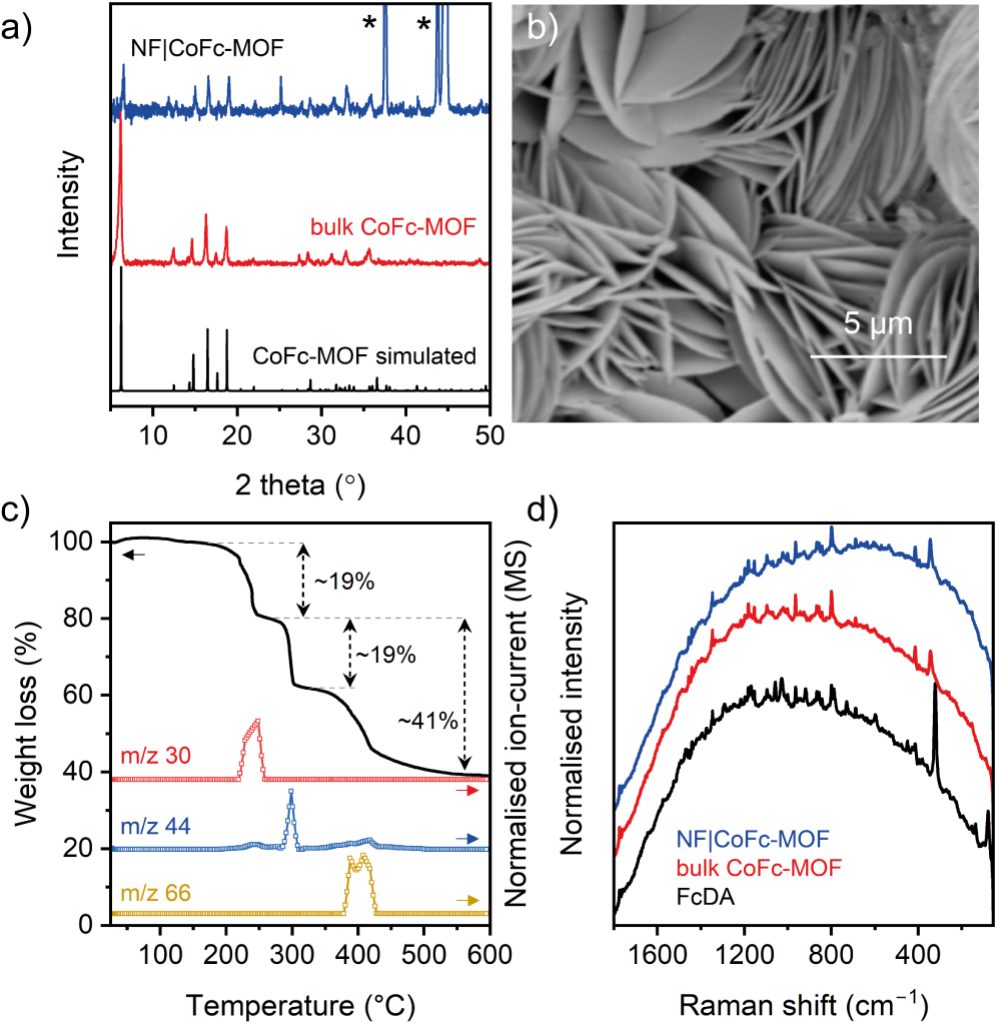
(a) Powder X-ray diffraction pattern of NF|CoFc-MOF (blue), bulk CoFc-MOF (red), and simulated CoFc-MOF (black) based on the optimized structure. The peaks marked with an asterisk in NF|CoFc-MOF originate from the NF substrate. (b) SEM image of NF|CoFc-MOF. (c) TGA of CoFc-MOF with simultaneous analysis of evolved gas using mass spectroscopy. (d) Raman spectra of FcDA linker (black), bulk CoFc-MOF (red), and NF|CoFc-MOF (blue).

CoFc-MOF nanosheets were directly grown on nickel foam electrodes via facile solvothermal synthesis (Figure 1). During synthesis, CoCl_2_ and FcDA were dissolved in aqueous DMF, and a clean Ni foam (NF) was immersed in the precursor solution. After heating the closed system at 120 °C, a CoFc-MOF film was developed on the surface of the Ni foam (denoted as NF|CoFc-MOF) as demonstrated by the visible change of color to dark yellow-brown (Figure S1). The structure and morphology of NF|CoFc-MOF and the bulk CoFc-MOF were characterized with a number of techniques. The crystalline phase of CoFc-MOF (bulk and film) was investigated using powder X-ray diffraction (PXRD). As shown in Figure 2a, the experimental PXRD pattern of CoFc-MOF is consistent with the simulated diffraction pattern, implying the successful fabrication of MOF. The crystal structure of CoFc-MOF was optimized using DFT based on previously reported ZnFc-MOF structure to yield a layered monoclinic structure with a slight modification of the unit cell parameters (*a* = 28.7 Å, *b* = 3.2 Å, *c* = 6.2 Å, α = γ = 90°, β = 98.4°).^32^ The optimized structure is shown in Figure 1. The CoFc-MOF structure is composed of infinite chains of CoO_6_ octahedra, forming the inorganic building unit. Four of the six coordinated oxygen atoms are part of the carboxylate groups from FcDA linkers. Two bridging hydroxide ions occupy the other positions which connect the octahedra in corner sharing fashion on the (200) plane to form the coordination polymer. The partial density of states (PDOS), as shown in Figure S2, shows a significant electron density around the Fermi energy (*E*_F_). This electron density clearly shows a metal-like electronic structure of CoFc-MOF. The morphology of bulk CoFc-MOF and film grown on NF was characterized using scanning electron microscopy (SEM), as shown in Figures 2b and S3–S4. The densely packed, vertically grown nanosheets display uniform coverage over the NF skeleton. The morphology of the bulk CoFc-MOF is similar to that grown on NF (Figure S3). The energy-dispersive X-ray spectroscopy (EDS) map of the material showed a matching uniform distribution of O, Fe, and cobalt within the particles (Figures S5 and S6). However, a slightly higher than expected Fe:Co ratio (1.5:1) was obtained from EDS analysis. The lateral size and thickness of the sheets were estimated to be ∼10 μm and ∼140 nm, respectively, using atomic force microscopy (AFM) (Figure S7). For comparison, analogous MOF films with an FcDA linker and either nickel- or iron-based nodes (NiFc-MOF and FeFc-MOF) were grown on Ni foam electrodes using the same solvothermal method by substituting CoCl_2_ with NiCl_2_ or FeCl_2_ (Figure S8).

The chemical compositions and the chemical environment of CoFc-MOF were characterized by infrared (IR), Raman, ^1^H NMR, inductively coupled plasma (ICP), and X-ray photoelectron spectroscopy (XPS). The IR C=O stretching band at 1670 cm^–1^ from carboxylic acid in FcDA disappeared in the CoFc-MOF and a new band appeared at 1575 cm^–1^, indicating the formation of carboxylate that coordinates the cobalt centers (Figure S9). The stretching bands at ∼1030 and ∼1390 cm^–1^ can be attributed to the breathing mode and asymmetric stretching mode, respectively, for the Cp ring in ferrocene. The Raman spectra of bulk CoFc-MOF and the film grown on NF showed characteristic peaks originating from FcDA ligands at 416, 800, 864, 965, 1026, 1095, 1181, and 1347 cm^–1^ (Figure 2d) that can be attributed to various vibrational modes of the ferrocene structure.^33^ The ring external mode for Fc appeared at 341 cm^–1^ for CoFc-MOF and shifted to a higher energy in comparison to the uncoordinated FcDA ligand (324 cm^–1^). The thermal stability of CoFc-MOF was probed using thermogravimetric analysis (TGA) and differential scanning calorimetry (DSC) coupled to *in situ* evolved gas analysis with mass spectroscopy (MS) (Figures 2c and S10). The sample remained stable up to 200 °C, and upon further heating, three weight loss steps were observed at ∼230 °C, ∼300 °C, and ∼400 °C, with clear stability plateaus in between. Based on the MS data shown in Figure 2c, the three steps can be attributed to ∼19 wt % loss of DMF molecules at ∼230 °C, ∼19 wt % loss from decarboxylation of FcDA linkers at ∼300 °C, and ∼22 wt % loss from release of cyclopentene fragments and decomposition of ferrocene at ∼400 °C. Based on the molecular formula of [Co(OH)(FcDA)·DMF], the calculated weight loss values for the adsorbed solvent molecule and the decomposition of the framework are in good agreement with the observed values (Table S1). The structural integrity of the FcDA linkers in the MOF was confirmed by ^1^H NMR spectroscopy of the digested sample, which exhibited peaks at chemical shifts matching FcDA. By using an internal standard during ^1^H NMR, the loading of FcDA was determined to be 1.7 mmol g^–1^ (Figure S11), which was slightly lower than the theoretical value of 2.4 mmol g^–1^. ICP analysis of CoFc-MOF shows a 1:1 atomic ratio for Co:Fe and a slightly higher metal loading of 2.8 mmol of Co/Fe g^–1^.

The XPS spectra collected for CoFc-MOF are shown in Figures S12–S15. The cobalt region of the XPS spectra exhibited two main peaks at 780.9 and 796.8 eV, corresponding to 2p_3/2_ and 2p_1/2_ signals (Figure S13). The two satellite peaks at 786.3 and 803.0 eV are consistent with Co^2+^ paramagnetic species.^34−36^ The Fe 2p region shows two main peaks at 711.3 and 725.1 eV corresponding to 2p_3/2_ and 2p_1/2_ signals (Figure S14).^37,38^ The peak positions suggest that the surface ferrocene units are mostly in the oxidized ferrocenium form. Deconvolution of the signals reveals the satellite peaks at 719.4 and 733.4 eV, corresponding to 2p_3/2_ and 2p_1/2_ of Fe^3+^. The energy separation between the 2p_3/2_ signal (711.3 eV) and satellite signal (719.3 eV) was 8 eV, which is consistent with Fe^3+^ species and is significantly higher than the value expected for Fe^2+^ (∼5 eV).^38^ Notably, a set of peaks at 708.3 and 720.8 eV, corresponding to 2p_3/2_ and 2p_1/2_ of Fe^2+^, can be observed in the deconvoluted spectra, suggesting the presence of minor nonoxidized ferrocene units. This result contrasts with the Fe 2p XPS data reported for Ni-based Fc MOFs that showed divalent Fe in ferrocene linkers.^28,30^ Fe^3+^ compounds often display complex multiplet-split Fe 2p spectra, leading to asymmetric peak shapes.^36,39^ The peak at ∼714 eV in the deconvoluted spectrum of CoFc-MOF is, therefore, tentatively attributed to the multiplet structure of Fe^3+^ ions. Deconvolution of high-resolution O 1s XP spectrum of CoFc-MOF revealed three components at 530.1, 531.5, and 532.4 eV that can be attributed to lattice oxygen (M–O), C–O, and C=O (from carboxylate), respectively (Figure S15).^30^ It should be noted that the O 1s spectrum contains contributions from the FTO substrate.

### Electrochemical Testing

**Figure 3:**
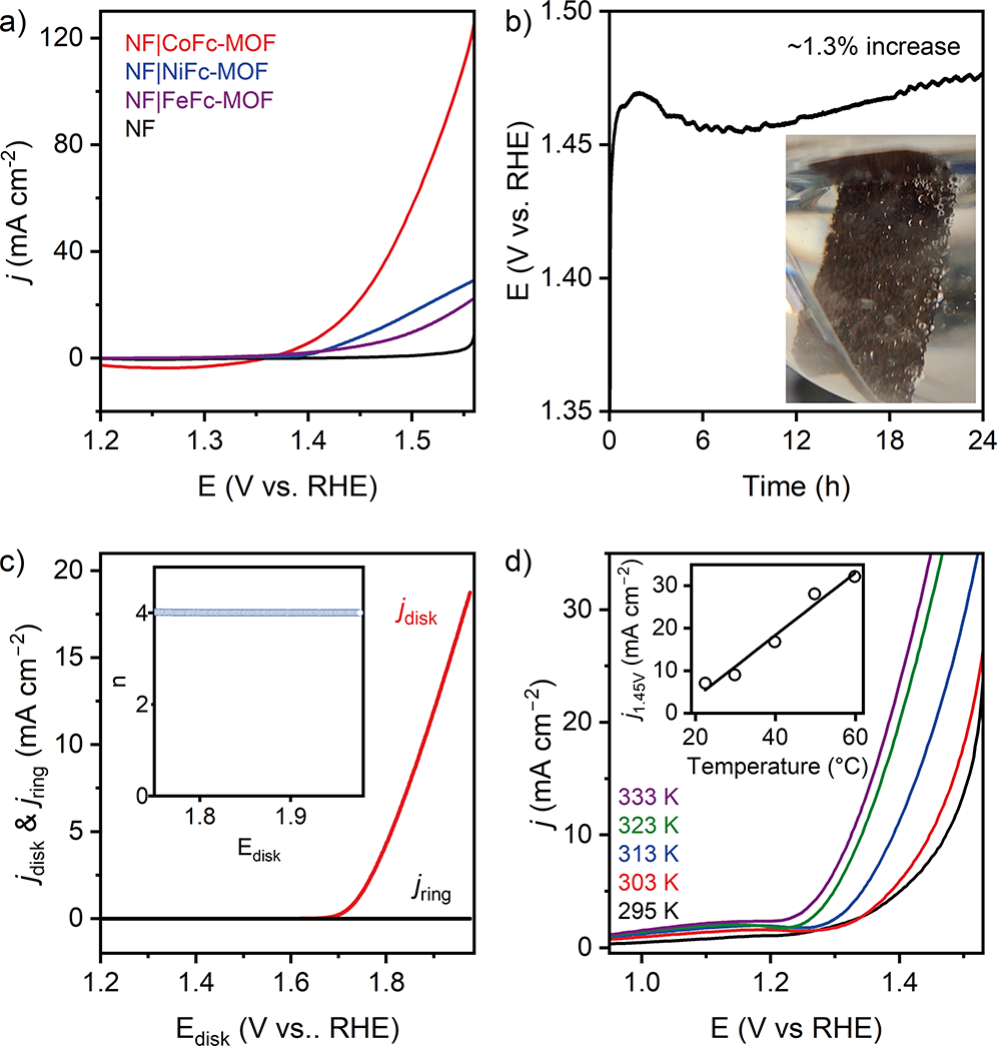
Electrochemical studies of CoFc-MOF films: (a) reverse LSV curves of NF|CoFc-MOF, NF|NiFc-MOF, NF|FeFc-MOF, and NF in 1 M KOH (potential scanned from high to low potential). (b) Chronopotentiometry of NF|CoFc-MOF at 10 mA cm^–2^ in 1 M KOH; the inset shows the O_2_ bubbles on the electrode. (c) Rotating ring-disk electrode (RRDE) voltammogram obtained for NF|CoFc-MOF in 0.1 M KOH (1600 rpm, 10 mV s^–1^). The ring potential was set at +0.4 V for the oxygen reduction reaction. The inset shows the corresponding electron transfer number (n) as a function of the applied potential. (d) LSV curves of NF|CoFc-MOF at various temperatures. The inset shows the linear correlation between the current density at 1.45 V and the temperature.

The OER performance of the CoFc-MOF-coated nickel foam (NF) electrode (NF|CoFc-MOF) was evaluated in 1 M KOH using a two-compartment H-cell separated by an anion exchange membrane. A standard three-electrode configuration was employed with NF|CoFc-MOF as the working electrode, blank NF as the counter electrode, and Ag/AgCl reference electrode. All data was acquired without *iR*-correction, and the potentials are reported against RHE. The geometric area of the electrodes was used for calculating current densities. As shown in Figure 3a, linear sweep voltammogram (LSV) shows the electrocatalytic water oxidation reaction by NF|CoFc-MOF, with an overpotential (η) of 190 mV at 10 mA cm^–2^. During LSV, the potential was scanned in the reverse direction, from high to low potential, to avoid the overlap between peaks for oxidation of metal centers and water. In comparison, analogous FcDA linker-based MOFs containing nickel and iron nodes (NF|NiFc-MOF and NF|FeFc-MOF, respectively) displayed lower activity with overpotentials of 233 mV for NiFc-MOF and 272 mV for FeFc-MOF at 10 MA cm^–2^.^30^ Tafel analysis of the LSV of NF|CoFc-MOF gave a slope of 65 mV dec^–1^, which is lower than those of NF|FeFc-MOF (120 mV dec^–1^) and blank NF (97 mV dec^–1^), but higher than those of NF|NiFc-MOF (49 mV dec^–1^) (Figure S16). A slightly higher Tafel slope for CoFc-MOF compared to NiFc-MOF suggests slower kinetics, which can be attributed to the different nature of the active sites (Co vs Ni) and/or particle morphology. The catalytic activity of the MOF-coated electrode is directly influenced by the electrochemical active surface area (ECSA) as it can potentially serve as a proxy for the number of accessible active sites. ECSA is proportional to the double-layer capacitance (*C*_dl_), which can be determined from variable scan rate CVs in the non-Faradaic region (Figure S17). From the slope of the linear fit of scan rate versus capacitive current plots, *C*_dl_ values for NF and NF|CoFc-MOF were determined to be 1.15 and 4.71 mF cm^–2^, respectively. This increase in *C*_dl_ is consistent with the sheet-like morphology of CoFc-MOF that results in more exposed active sites to the electrolyte.

The longevity of the NF|CoFc-MOF electrodes under operating conditions was demonstrated by chronopotentiometry at 10 mA cm^–2^, which showed a steady voltage response at ∼1.45 V for 24 h (η = 220 mV) (Figure 3b). This indicates the robustness of the electrode, capable of delivering sustained OER activity. An average electron transfer number (n) during electrocatalysis was determined to be 3.97 using rotating ring-disk electrode measurement (Figure 3c), implying that the water oxidation on NF|CoFc-MOF electrode surface follows a four-electron pathway (4OH^–^ → 2H_2_O + O_2_ + 4e^–^).^40^ The evolution of the structure of O_2_ during chronopotentiometry was supported by the bubbles observed at the electrode surface (Figure 3b). The OER activity of the NF|CoFc-MOF was further investigated at variable cell temperatures. LSVs were recorded at the cell temperatures between 295 and 333 K, while keeping other conditions unchanged. On increasing the cell temperature from 295 to 333 K, a linear increase in the current density (*j*) at a fixed potential was observed. Figure 3d (inset) shows the trend at 1.45 V, a relatively low overpotential of 220 mV, where the OER is primarily under kinetic control, and mass transfer limitations are assumed to be small. The enhanced OER activity observed at elevated temperatures can be caused by a combination of factors, including improved mass transportation, facile desorption of oxygen bubbles from the electrode, faster electron transfer rate, and lowering of thermodynamic water oxidation potential.^41,42^

**Figure 4:**
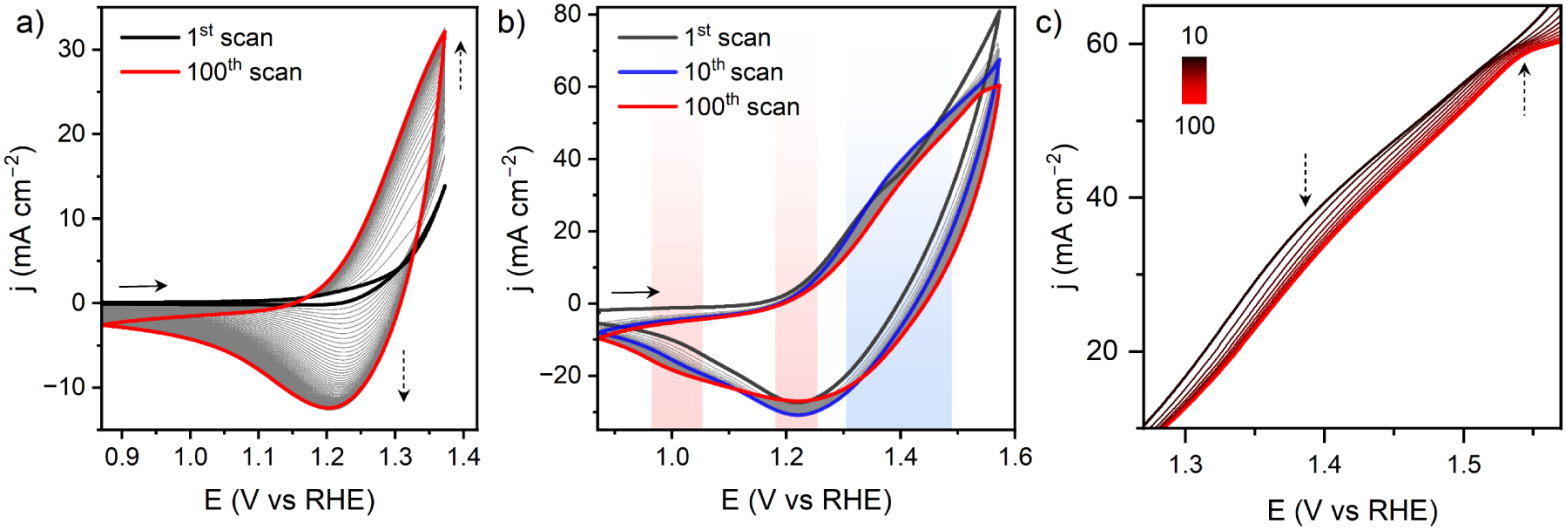
Continuous cyclic voltammograms (100 scans) recorded for NF|CoFc-MOF in 1 M KOH at 10 mV s ^–1^ over potential range (a) 0.87–1.37 V (catalytic-onset region) and (b) 0.87–1.57 V (postcatalytic region). (c) Zoom of the forward scans in (b), demonstrating the appearance of the anodic peak at ∼1.52 V after 100 scans.

The electrochemical stability of CoFc-MOF and potential structural change at the electrochemical double layer were investigated by cyclic voltammetry (CV) treatment of NF|CoFc-MOF for 100 cycles in 1 M KOH. The material was first scanned in the non-Faradaic region, 0.87–0.97 V, where the electrode displayed typical capacitor behavior originating from the adsorption and desorption of hydroxide ions within the double-layer region (Figure S18). With an increasing number of scans, the voltammograms became flatter, but the capacitive current density remained largely unchanged. The scan rate dependence of the capacitive current revealed only a small increase in electrochemical surface area (ECSA) from 1st to 100th cycles, which can be attributed to the morphological evolution of CoFc-MOF (Figure S19). Interestingly, electrochemical impedance spectroscopy (EIS) recorded at 0.87 V showed a steady increase in the phase angle of the straight line in the midfrequency region, which is primarily controlled by the morphology and surface area of the materials (Figure S20a).^43^ Deviation from the Warburg diffusion element (phase angle >45°) is consistent with a high OH^–^ diffusion rate through the porous structure, which increased with the number of scans. This implies the formation of a microstructure with high porosity and a better OH^–^ adsorption capability. Upon widening the scanning potential window to 0.87–1.37 V (catalytic-onset region), a distinct partially reversible redox process evolved at ∼1.28 V, which overlapped with the onset of OER (Figure 4a). Increasing anodic and cathodic currents with the number of scans demonstrates continuous exposure of more accessible Co sites within the CoFc-MOF electrode, suggesting restructuring at the molecular level. The redox process can be attributed to Co^3+/2+^ couple, and we speculate that the restructuring led to the formation of cobalt oxyhydroxide. Over 100 CV cycles, the phase angle of the linear region of EIS remained largely unchanged, which suggests that the microstructure formed during the initial conditioning of the electrode (CV scans in 0.87–0.97 V) remained stable during the onset of electrocatalytic OER (Figure S20b). A subtle change in the shape of the voltammogram was observed when the electrode was scanned at further oxidizing potential (0.87–1.57 V), beyond the onset of OER (Figure 4b). The anodic wave at ∼1.35 V incrementally shifted to a more positive potential from the 1st to 10th scan (blue shaded region in Figure 4b), while the cathodic current during the return scan gradually increased (black and blue traces in Figure 4b). Further CV scans led to the appearance of a well-defined anodic peak at ∼1.52 V (Figure 4c) with the corresponding decrease of the reductive peak current at ∼1.22 V and increase of the current at ∼1.02 V (red shaded regions in Figure 4b). The EIS at 0.87 V remained largely unchanged over the CV scans (Figure S20c). The combined data suggests evolution of a new active species during catalysis, while the porous microstructure of the material was retained. Interestingly, the ECSA estimated from *C*_dl_ after 100 CV scans demonstrated a steady increase when the electrode was scanned over wider potential windows, from the capacitive region (0.87–0.97 V) to catalytic-onset region (0.87–1.37 V) and post-catalytic region (0.87–1.57 V) (Figure S21). This change in ECSA implies potential restructuring of CoFc-MOF during electrochemical treatment and the OER.

### Postcatalysis Characterization

Post-catalysis NF|CoFc-MOF electrodes showed a visible darkening of color, suggesting that the material undergoes change in its electronic structure (Figure S1). During electrolysis, the combination of alkaline electrolyte and strongly oxidizing conditions can induce structural changes in the catalyst, affecting its morphology, crystallinity, and microstructure. To gain an in-depth understanding, a range of *in situ* and *ex situ* characterizations were performed on the CoFc-MOF under operating conditions and post-catalysis. PXRD pattern of the post-catalysis electrode showed a complete loss of the characteristic peaks for the CoFc framework and a new broad reflection in the 2θ range 25–45° (Figure S22a,b), which suggests a structural transformation of CoFc-MOF. Notably, the PXRD pattern of only KOH-treated CoFc-MOF also revealed a loss of the characteristic MOF peaks with the appearance of new reflection peaks at 19°, 32°, 38°, 52°, 58°, and 62°, which points toward the formation of β-Co(OH)_2_ phase in the electrolyte (Figures S22 and S23).^44^ A broad peak at ∼10° with low intensity can be assigned to α-Co(OH)_2_ (Figure S23c). During electrolysis, Co(OH)_2_ likely undergoes further oxidation and structural changes, forming an active catalyst layer of metal oxyhydroxide with low crystallinity.

**Figure 5:**
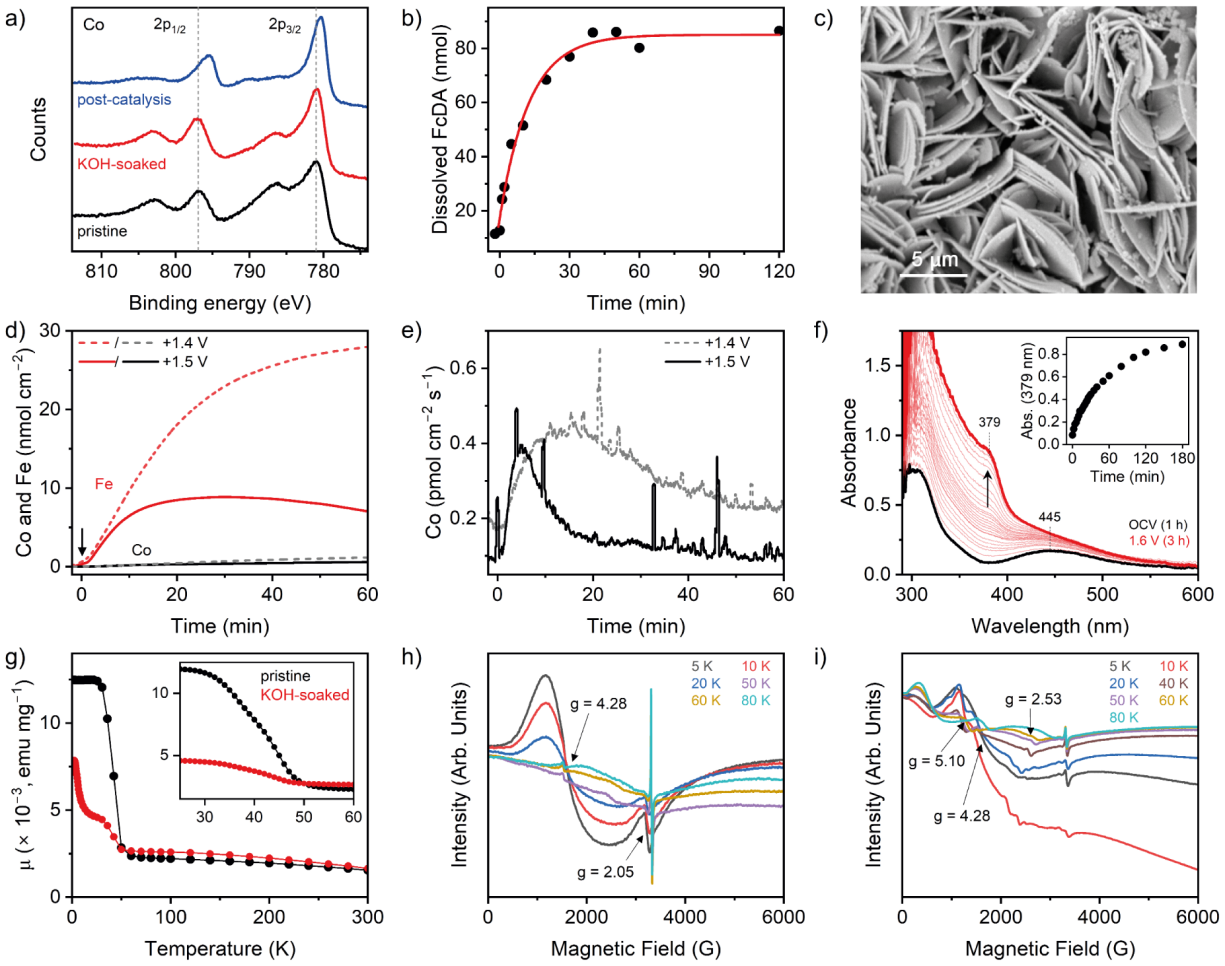
(a) Co 2p region of the XPS of pristine, electrolyte-soaked, and post-catalysis CoFc-MOF grown on FTO electrodes. (b) HPLC quantification of the FcDA released from NF|CoFc-MOF in the electrolyte during chronopotentiometry at 10 mA cm^–2^. The red trace shows an exponential fit of the data. (c) SEM image of the post-catalysis electrode. (d) *In situ* ICP-MS measurement to quantify the amount of cobalt and iron in solution during controlled potential electrolysis under a continuous flow of 0.01 M KOH solution. The black arrow shows the start of electrolysis. (e) *In situ* ICP-MS analysis of cobalt in the electrolyte, showing the rate of leaching of cobalt. (f) *In situ* UV–vis spectroelectrochemistry of the electrolyte during 3 h chronoamperometry with CoFc-MOF electrode (loading ∼0.8 mg cm^–2^ on carbon paper) at +1.5 V (vs RHE). The blacktrace shows the pre-electrolysis UV–vis spectra of the electrolyte after equilibrating the electrode for 1 h. The red trace shows the UV–vis spectra of the electrolyte after 3 h chronoamperometry. The inset shows the increase of absorbance at 379 nm with time. (g) Variable-temperature magnetic moment data for pristine and KOH-soaked CoFc-MOF. The inset shows the ferromagnetic transition for the two samples. (h, i) Variable-temperature X-band EPR spectra of pristine (h) and KOH-soaked (i) CoFc-MOF in the temperature range 5–80 K. The spectra were recorded using a microwave frequency of 9.37 GHz and a microwave power of 2 mW.

XPS spectrum of KOH-treated MOF showed that the binding energies of Co 2p_3/2_ and 2p_1/2_ peaks remain largely unchanged (Figures 5a and S13). The prominent satellite peaks at ∼786 and ∼802 eV are consistent with the high spin Co^2+^ state in Co(OH)_2_.^34−36,45,46^ In contrast, the postcatalysis electrode showed a clear shift of the Co 2p peaks to lower energy (780.4 and 795.5 eV) and a decrease in the intensity of the satellite peaks. These observations point to the Co being either completely or partially in a low-spin Co^3+^ state,^47^ and based on the peak positions, it can be attributed to a cobalt oxyhydroxide species.^48,49^ Deconvolution of the higher-intensity 2p_3/2_ peak showed another fitted peak at 781.8 eV for the postcatalysis sample, which was shifted to lower binding energy compared to the pristine and KOH-soaked samples (Figure S13). The Fe 2p XPS spectra of the KOH-soaked and post-catalysis electrode displayed an increase in the intensity of the shakeup satellite peak at ∼718.7 eV that can be attributed to the formation of Fe^3+^–OH species on the surface (Figure S14). However, the binding energies for the 2p_3/2_ and 2p_1/2_ peaks remained almost unaffected, suggesting a similar valence state for the two samples. The broadening of Fe 2p_3/2_ peaks in KOH-soaked and postcatalysis electrodes compared to the pristine CoFc-MOF further supports the transformation of low-spin mixed-valence Fe^2+^/Fe^3+^ ferrocene centers to high-spin Fe^3+^-(oxy)hydroxide species.^36,39^ Analysis of O 1s indicates an increase in the M–O species (530.0 eV) after catalysis, which is in accordance with the PXRD results (Figure S15). The carbon region of the XPS spectrum showed no obvious difference post-catalysis.

SEM images of the post-catalysis NF|CoFc-MOF electrode revealed that the overall morphology of densely packed sheets was maintained, but the exposed surface showed deposits of smaller aggregates on the sheets (Figure 5c). EDS analysis of the post-catalysis electrode showed an increase in the surface concentration of Fe and Co, but the Fe:Co atomic ratio (1.4:1) was close to that in pre-catalysis MOF. Furthermore, a notable decrease in carbon content and an increase in Ni and O content were observed (Table S2). The increase in O content is consistent with the formation of metal hydroxide/oxyhydroxide species that likely play a key role in electrocatalysis. The increased Ni content at the surface can be caused by a more exposed NF substrate and/or migration of Ni^2+^ from the substrate (NF) into the active catalyst layer. The loss of carbon from the surface can be explained by leaching of FcDA linkers during catalysis. To confirm this, the electrolyte was monitored using HPLC to quantify the amount of linker released in the solution during electrolysis. As shown in Figure 5b, the FcDA linker started leaching in the electrolyte upon application of the oxidative potential (*t* = 0 min). The concentration of linker plateaued at ∼40 min, and the amount of FcDA in solution after 2 h electrolysis corresponded to <1% weight loss of the MOF-film. The kinetic data fitted an exponential process. Pre-electrolysis equilibration of the electrode showed a negligible concentration of FcDA in the electrolyte (∼0.01 μmol), but the leaching of the linker became prominent after the oxidative potential was applied. Raman spectrum of the postcatalysis electrode showed a mixture of strong vibration bands in the 480–690 cm^–1^ range that can be correlated to the formation of metal oxyhydroxide species [Co(Fe)OOH] on the surface (Figure S24).^50−52^ Furthermore, ATR-IR of the evolved electrode material showed a loss of the characteristic vibrations for CoFc-MOF and a broad peak at ∼500 cm^–1^, again suggesting the formation of metal oxyhydroxide (Figure S9).

The restructuring of the catalyst was further supported by *in situ* ICP MS analysis under continuous flow and applied potential. CoFc-MOF was deposited on carbon paper and mounted in an *in situ* flow cell while the electrolyte (0.01 M KOH) was constantly passed through the cell and fed into the ICP-MS. Carbon paper support minimized the physical detachment of CoFc-MOF and ensured that the Co and Fe detected in solution were derived only from chemical leaching and/or electrochemical decomposition. The stability of the material was probed at +1.4 V and +1.5 V, at electrolyte flow rates of 0.49 and 0.44 mL min^–1^, respectively. As shown in Figure 5d, the Fe signal in the electrolyte is considerably higher than the Co signal at both potentials, indicating a higher loss of Fe species from the electrode. Figure 5e shows that the rate of Co release (detected by ICP) increased with electrolysis time before reaching a maximum rate (∼5 and ∼14 min at +1.5 V and +1.4 V, respectively), followed by a slow decay of the rate. This result is consistent with the HPLC data and hints toward a structural reconstruction at the electrode surface during the initial few minutes of the electrolysis when FcDA linkers are released into the electrolyte and a cobalt-rich active catalyst layer is formed. Release of FcDA in the electrolyte was further supported by *in situ* UV–vis spectroelectrochemistry of the electrolyte (Figures 5f and S25). As shown in Figure 5f, the pre-electrolysis spectrum of the electrolyte showed clear peaks at 306 and 445 nm that can be attributed to dissolution of FcDA during the 1 h equilibration of the electrode in 1 M KOH (Figure S25a). The concentration of FcDA in the electrolyte showed minimal change upon incremental increase of the applied potential from 1.1 to 1.5 V (Figure S25b,c). However, during prolonged chronoamperometry at 1.5 V, the 445 nm band gradually disappeared with the concomitant appearance of a new peak at ∼379 nm (Figure 5f). This can be tentatively attributed to the oxidation of FcDA to ferrocenium species and its subsequent decomposition in the presence of ambient air and water.

The structural changes during KOH (1 M) treatment were further investigated by SQUID magnetometry and electron paramagnetic resonance (EPR) techniques on pristine and KOH-soaked CoFc-MOF. As shown in Figure 5g, the magnetic moment of pristine CoFc-MOF displayed a monotonical increase in magnetic susceptibility with decreasing temperature from 295 K, followed by a sudden jump below ∼50 K, which then plateaued at <25 K. This sudden jump in susceptibility is consistent with a ferromagnetic transition. The KOH-soaked MOF also displays a ferromagnetic transition at a similar Curie temperature (*T*_C_ ∼ 45 K), but the magnetic susceptibility of the KOH-soaked MOF continued to increase at low temperatures. The variable-field magnetization data collected at 300 K revealed a similar correlation (no saturation of magnetization at 300 K) for both pristine and KOH-soaked samples, but the KOH-soaked material displays a slightly higher magnetization than the pristine sample at fields between 1 and 7 T (Figure S26). The results are consistent with structural transformation of CoFc-MOF in the alkaline electrolyte. The two materials were further characterized by variable-temperature EPR measurements to probe the paramagnetic species, and the results are shown in Figures 5h,i and S27. At 5 K, pristine CoFc-MOF displayed a broad signal at g ∼ 4.28 and a weak EPR signal at ∼3350 G with *g* ∼ 2.05, which can be assigned to rhombic high-spin Co^2+^ (t_2g_^5^e_g_^2^, *S* = 3/2).^53,54^ As shown in Figure 5h, these signals gradually decrease in intensity (red and blue traces) with an increase in temperature and completely disappear at temperatures of 50 K and above. These observations are consistent with the ferromagnetic transition observed in SQUID measurements. At >60 K, a sharp EPR signal is observed at ∼3310 G for both pristine and KOH-soaked samples, that likely arises from oxygen vacancies.^55,56^ However, other possibilities like signals arising from Fe(II)/Fe(III) center cannot be ruled out. The assignment of individual EPR transitions to the specific metal ion and/or spins (integer/noninteger spins) warrants simulations and measurements at higher frequencies to “unpack” the overlapping signals, which are currently in progress. The room-temperature EPR spectrum of pristine CoFc-MOF featured a broad peak, suggesting an intrinsic paramagnetic signal originating from Co^2+^ and Fe(II)/Fe(III) species (Figure S27). The KOH-soaked sample displayed a similar broad signal at room temperature, but the spectra recorded at low temperatures (5–60 K) showed significant differences compared to the pristine sample (Figure 5i) consistent with the results obtained from the SQUID magnetic measurements. At 5 K, the KOH-soaked material exhibited predominant EPR signals near zero field and between 1000 and 1500 G (*g* ∼ 4.28), assigned to high-spin Co^2+^ species. With increasing temperature (10 and 20 K; red and blue traces), these signals showed resolved structures (splitting of the broad EPR signal) with a decrease in intensity. When the temperature was increased further, these signals disappeared and a set of new transitions were observed at 500 G, 1400–1500 G, and 2500 G, respectively, with correspondingly different g values. This suggests plausibly a mixed population of paramagnetic species and spin states in the intermediate temperature range. This is consistent with the susceptibility data observed below 45 K (*T*_C_ ∼ 45 K) for the KOH-soaked CoFc-MOF materials, where two maxima are observed – one at ∼25 K and the other at 5 K. All these observations corroborate that alkali treatment altered the structure of CoFc-MOF.

**Figure 6:**
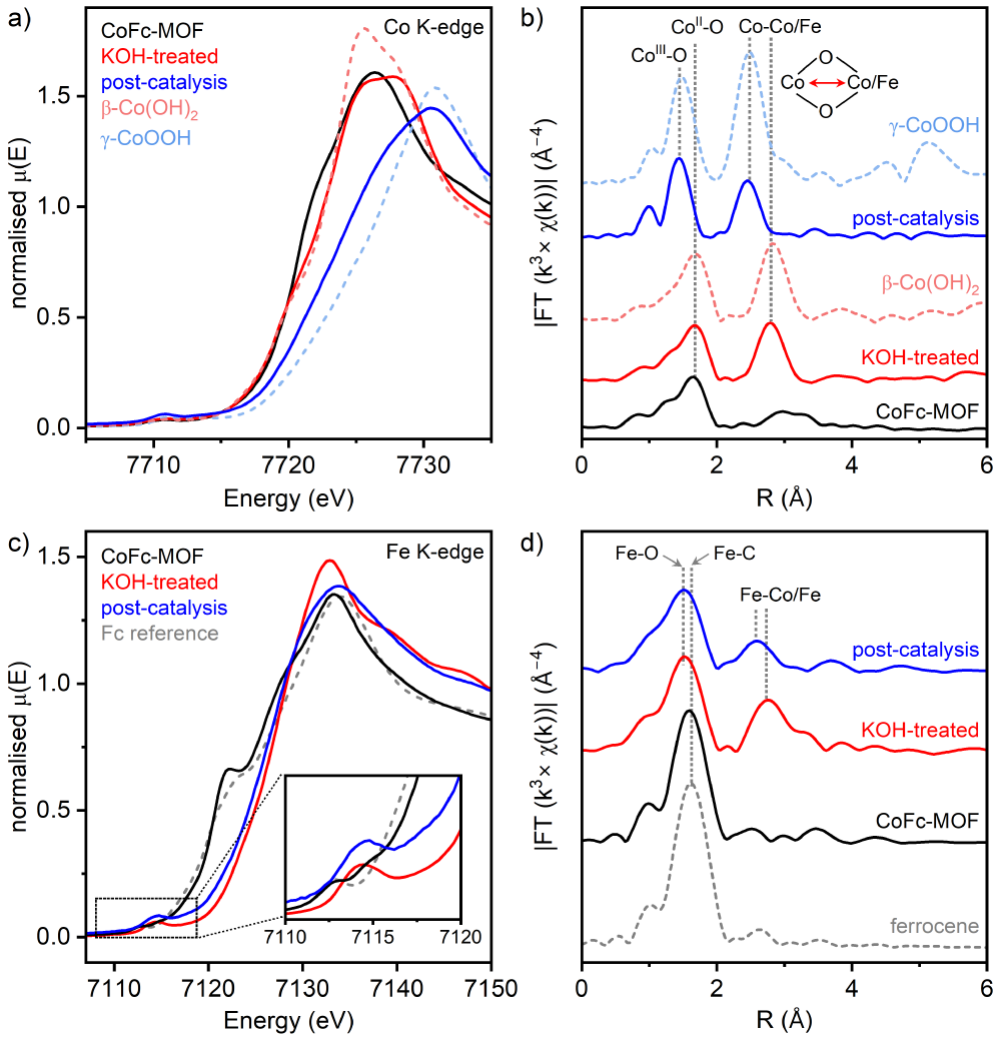
(a) Co K-edge XANES spectra and (b) Fourier-transformed *k*^3^-weighted EXAFS signals for pristine CoFc-MOF, KOH-treated CoFc-MOF, and post-catalysis material compared with two cobalt reference compounds. (c) Fe K-edge XANES spectra and (d) Fourier-transformed *k*^3^-weighted EXAFS signals for the same three materials and ferrocene reference.

Changes in the local electronic and atomic structures around the metal centers inside CoFc-MOF were investigated by X-ray absorption spectroscopy (XAS) measurements at the Co and Fe K-edges, including X-ray absorption near-edge structure (XANES) and extended X-ray absorption fine structure (EXAFS) analysis. Figures 6, S28, and S29 and Tables S3 and S4 summarize the XAS data collected with pristine CoFc-MOF, electrolyte-soaked CoFc-MOF, and the post-catalysis material. The Co K-edge XANES positions of pristine and soaked CoFc-MOF overlap closely with Co(OH)_2_, confirming the +2 valence state of Co. The post-catalysis sample shows a clear shift of the absorption edge (E_0_) to higher energy, demonstrating an increase in the Co oxidation state to +2.6 (Figure 6a).^49,57,58^ The Fourier transform of Co K-edge EXAFS (*k*^3^-weighted) spectrum of pristine CoFc-MOF showed a dominant peak at 1.65 Å corresponding to the nearest-neighbor (Co–O bond) contribution, and a broad peak at 3.0–3.3 Å assigned to Co–Co/Fe scattering paths (Figure 6b). The EXAFS data was fitted using the FEFF input based on the DFT-optimized molecular structure of CoFc-MOF, and the results are summarized in the SI (Figures S28 and S29, Tables S3 and S4). After KOH treatment, a new peak was observed at 2.80 Å, while the position of the first Co–O peak at 1.65 Å remained unchanged. The resulting features are similar to those of Co(OH)_2_ where the second peak originates from the M–M scattering paths between the metals of di-μ-oxo bridged [MO_6_] octahedra. The EXAFS data indicate that KOH-induced structural changes of CoFc-MOF had minimal impact on the first coordination shell (CoO_6_ octahedra) but led to a new Co–Co/Fe coordination shell from the formation of edge-sharing [MO_6_] units. EXAFS of the post-catalysis material demonstrated a shift of Co–O and Co–Co/Fe peaks from 1.65 to 1.44 Å and 2.80 to 2.45 Å, respectively, which is consistent with the bond length shrinkage from hydroxide to oxyhydroxide analogues, leading to shorter scattering paths.^59,60^ The resulting features are consistent with those of CoOOH, albeit with lower peak intensities, which suggests that the post-catalysis material has a comparable but more disordered local structure with a few defects in the overall lattice. The fitting of EXAFS data reveals two Co–O distances (first shell) at 1.87 and 1.97 Å with a total coordination number (CN) of 5.3, and two Co–Co/Fe distances (second shell) at 2.79 and 2.84 Å with a total coordination number of 5.2 (Table S3). A coordination number of 6 is expected for both shells in a perfectly ordered material with large layers, and the lower values derived from the fit suggest that the metal oxyhydroxide sample contains oxygen vacancies and smaller layer sizes.^61^

The Fe K-edge XANES of the pristine CoFc-MOF shows a pre-edge peak (∼7112 eV) assigned to a quadrupole allowed 1s → 3d e_1g_ transition, a shoulder on the rising absorption edge (∼7122 eV) attributed to a 1s → Cp (π*) transition in FcDA linkers, and an *E*_0_ value of 7121 eV (Figure 5c).^62^ In comparison, the leading portion of the edge for ferrocene reference is shifted to lower energy (Figure 5c inset, gray dashed trace), and the shoulder peak at ∼7123 eV has lower intensity. These features indicate that the bulk MOF contains oxidized FcDA linkers.^62^ Alkali treatment of the MOF resulted in a shift of the pre-edge feature to ∼7114 eV, a complete loss of the shoulder peak, and an increase in the *E*_0_ value to ∼7127 eV, suggesting a significant reconstruction of the local structure around Fe. We hypothesize that this change is related to the formation of metal hydroxide species. The post-catalysis material displayed a small red shift in the absorption edge (<1 eV decrease in *E*_0_) and a decrease in intensity of the main XANES absorption peak (Figure 5c, blue and red traces).

The Fe K-edge *k*^3^-weighted EXAFS spectrum of pristine CoFc-MOF is dominated by a single peak at 1.59 Å, assigned to the Fe–C coordination shell in ferrocenium linkers with a Fe–C bond distance of 2.07 Å (Figure 6d). After alkali treatment, the main peak shifted to a lower distance at 1.52 Å and a new peak appeared at 2.76 Å. This can be attributed to the formation of mixed metal Co/Fe-hydroxide, and the EXAFS data can be simulated by a Fe–O shell at 2.01 Å bond distance and a Fe–Co/Fe shell at 3.14 Å (Figure S27 and Table S4). Post-catalysis material demonstrated a nearly unchanged bond distance in the Fe–O shell with a small decrease in coordination number from 5.8 to 5.3. However, the second coordination shell exhibited a shortening of the Fe–Co/Fe distance to 2.98 Å and a decrease of the coordination number from 4.1 to 3.3, consistent with higher oxygen vacancies in the resulting Co/Fe-oxyhydroxide material. Based on XAS results, we propose that the CoFc-MOF electrocatalyst undergoes a dynamical structural reconstruction during the electrocatalysis, from the crystalline MOF to mixed metal Co/Fe-hydroxide in the resting state (in 1 M KOH electrolyte) and then to amorphous Co/Fe-oxyhydroxide [Co(Fe)OOH] under oxidizing conditions. This active catalyst provides enhanced water oxidation activity due to the increased number of active sites, high surface area, and tuned structural properties. It should be noted that when nickel foam is used as the substrate, the presence of nickel doping within the metal oxyhydroxide layer cannot be excluded, as shown by the EDS analysis of the post-catalysis electrodes.

## Conclusions

In summary, CoFc-MOF was synthesized on nickel foam via solvothermal method with the material displaying a 2D nanosheet morphology. The CoFc-MOF contains Co(II) centers at the node and oxidized ferrocenium linkers. Electrochemical investigation shows high activity of the NF|CoFc-MOF electrodes toward OER, but an in-depth characterization of the material demonstrated structural instability of the MOF under operating conditions, leading to *in situ* formation of metal oxyhydroxides that served as the real active sites. *Ex situ* XAS and XPS characterization of the material suggest an irreversible structural transformation of CoFc-MOF to Co(II)/Fe(III)-hydroxide in alkaline electrolyte via replacement of coordinated organic linkers by OH^–^, which was supported by HPLC analysis of the electrolyte as well as EPR and magnetometry characterization of the materials. At mildly oxidizing potentials in the precatalytic region (<1.3 V), the potential sweep disrupts the Co^2+^–linker coordination bonds and exposes more accessible Co^2+^ sites with increasing cycle numbers. Electrolysis at higher potential during the OER leads to further oxidation of the exposed Co^2+^ sites to their higher valent form, Co(III)/Fe(III)-oxyhydroxide. The presence of oxygen vacancies in the surface-evolved mixed metal oxyhydroxide is demonstrated by the fitting of the EXAFS data, which likely facilitates the adsorption of substrates at the Co active sites and promotes OER. The nanosheet morphology of the MOF regulates the nanostructuring of the active catalyst phase, in which the Fe(III) contribution comes from the ferrocene linkers. The results demonstrate that the electrocatalytic data obtained using MOF-based electrodes cannot be automatically attributed to the active sites embedded in the MOF architecture without evaluating its structural integrity under operating conditions. However, this work highlights the potential of using MOFs as pre-catalysts to exploit their intrinsic electronic properties and porosity in electrocatalyst design.

## Supporting Information Available

Details of characterization techniques and additional figures and tables for DFT calculation, SEM, AFM, PXRD, ATR-IR, NMR, TGA-DSC, XPS, Raman, electrochemical tests, EIS, SQUID, EPR, and XAS, as described in the text (PDF)
