# Supplementary material for: Structural Reconstruction of a Cobalt- and Ferrocene-Based Metal–Organic Framework during the Electrochemical Oxygen Evolution Reaction
Source: ACS Appl Mater Interfaces. 2024 Jul 23;16(31):40814–24. doi: 10.1021/acsami.4c03262 (PMC11310903; doi:10.1021/acsami.4c03262)
Supplement: Supplementary file 1 — am4c03262_si_001.pdf [file am4c03262_si_001.pdf]

Supporting Information

Structural reconstruction of a cobalt and ferrocene-based metal-organic framework during electrochemical oxygen evolution reaction

Thomas Doughty,[a] Andrea Zingl,[b] Maximilian Wünschek,[b] Christian M. Pichler,[b,c] Matthew B. Watkins,[d] Souvik Roy[a*]

[a] School of Chemistry, University of Lincoln, Green Lane, Lincoln, LN6 7DL, UK

[b] Institute of applied Physics, TU Vienna, Wiedner Hauptstraße 8-10, 1040 Vienna, Austriaa

[c] Centre of electrochemical and surface technology, Viktor Kaplan Straße 2, 2700 Wiener Neustadt, Austria

[d] School of Mathematics and Physics, University of Lincoln, Brayford Pool, Lincoln LN67TS, United Kingdom

Email: sroy@lincoln.ac.uk

Characterisation Techniques

The morphology of the sample was characterised using scanning electron microscopy (SEM, Tescan Essence) with energy-dispersive X-ray spectroscopy (EDX). Raman measurements were performed using a Bruker Senterra benchtop Raman microscope using 785 nm laser excitation and a 20× objective lens. The spectrometer was calibrated using a polystyrene standard. Raman spectra were collected over the 50-2630 cm^{-1} spectral range at 1 mW power with 60 s exposure time and 5 accumulations. Fourier-transform infrared spectroscopy (FTIR) was performed on a Bruker Alpha II using ATR accessory. Powder X-ray diffraction data was collected on a Bruker D8 Discover using a Cu Kα radiation source (λ=1.54178 Å). Thermal analysis was performed on Netzsch STA 449 F3 model attached with mass spectrometer QMS403. During the measurements, the purge gas flow (N_2) and protective gas flow (N_2) were 50 mL min^{-1} and 20 ml min^{-1}, respectively. The samples were heated from 30 °C to 600 °C at a rate of 10 °C min^{-1} with simultaneous mass spec analysis of the evolved gas. X-ray photoelectron spectroscopy (XPS) was performed on a Thermo Fisher Scientific K-alpha+ spectrometer. Samples were analysed using a micro-focused monochromatic Al x-ray source over an area of approximately 200 microns. Data were recorded at pass energies of 150 eV for survey scans and 40 eV for a high-resolution scan with 1 eV and 0.1 eV step sizes, respectively. Data analysis was performed in CasaXPS using a Shirley type background and Scofield cross sections, with an energy dependence of –0.6. ICP analysis was performed on a Thermo Scientific iCAP 7000 series ICP-OES Spectrometer, using the Qutegra software. Atomic Force Microscopy (AFM) was performed on an AFM Workshop HR-AFM. The images were processed and analysed using Gwyddion. X Band (9.4GHz) continuous Wave Electron Paramagnetic Resonance (EPR) measurements were carried out using a Bruker EMXPlus Spectrometer equipped with a Bruker ER4122-SHQ resonator. Cryogenic temperatures were achieved using a ColdEdge Stinger closed-cycle cryocooler mated to an Oxford Instruments ESR900 cryostat. Temperature maintenance and control was handled via an Oxford Instruments MercuryITC.

Electrochemical measurements: Electrochemical tests were carried out in 1M KOH using a three-electrode system on a BioLogic VSP potentiostat. A Ag|AgCl|KCl (satd.) reference electrode (CHI111) was used for all experiments. Pt-mesh and NF were used as the counter electrode for testing with FTO and NF, respectively. The Ag|AgCl|KCl (satd.) reference electrode was regularly calibrated against a master reference electrode and any drift in the potential (E') of the Ag|AgCl reference was corrected using the equation:

$E_{corrected}$ (V vs Ag|AgCl) = E (V vs Ag|AgCl) + E'

The reversible hydrogen electrode (RHE) potentials were obtained with the equation:

E (V vs RHE) = 0.197 + $E_{corrected}$ (V vs Ag|AgCl) + 0.059 × pH

Linear sweep voltammetry (LSV) measurements were performed at 10 mV s^{-1} using the reverse method, to avoid overlapping of oxidation peaks with the metal ion and water. Overpotentials (η) are calculated using the equation:

$\eta = E_{RHE} - 1.23V$

Tafel slopes were calculated from an LSV performed at 10 mV s^{-1} utilising the Tafel equation: $\eta = b \log(j) + a$; where η denotes overpotential, j denotes current density, a is a constant and b is the Tafel slope. Chronopotentiometry (CP) was performed at 10 mA cm^{-2} to evaluate the stability of the CoFc MOF. The variation in potential over the course of 24 h chronopotentiometry was determined to be ~1.3%. During chronopotentiometry, the potential stabilised at ~1.470 V (vs. RHE) after ~1.4 h, followed by a gradual decrease to ~1.455 V after 8h. Afterwards, the potential slowly increased to 1.475 V after 24 h electrolysis. The increase in potential was ~20 mV from 8h to 24 h corresponding to a ~1.3% increase.

The electrochemical active surface area (ECSA) was calculated by recording CVs at variable scan rates (5-100 mv s^{-1}) in the non-faradaic region from 0.75 to 0.80 V (vs RHE). The double-layer capacitance (C_{dl}) is obtained by plotting the capacitive currents at 0.775 V, with the C_{dl} equalling half the gradient.

XAS measurements: XAS data was collected at beamline B18 at the Diamond Light Source (beamtime number SP-34632-1). Pellets (13 mm) for pristine and KOH-soaked CoFc-MOF were prepared by homogeneously mixing MOF powders with an inert cellulose matrix and mounted on a sample holder using Kapton tape. For the post-catalysis sample, 0.25 mL catalyst ink containing CoFc-MOF (16 mg CoFc-MOF dispersed in 2 mL IPA containing 0.04 mL 5 wt% Nafion solution using 30 min sonication) was drop casted on 1×1 cm^2 carbon paper electrode (AvCarb GDS2120), which was subjected to 6 h controlled potential electrolysis at 1.5 V. The electrode was cut into four 0.25 cm^2 squares and stacked using Kapton tape for XAS measurements. $Co(OH)_2$ (97%, Thermo Scientific Chemicals) and CoOOH (synthesised using a literature method)[1] were used as the cobalt standards and Ferrocene as the iron standard. The PXRD patterns of $Co(OH)_2$ and CoOOH are shown in Figure S23. Measurements of the pellets were performed in transmission mode (3 scans), while the post-catalysis electrode was measured in fluorescence mode (6 scans). XAS data processing and EXAFS analysis were performed using Athena and Artemis software, respectively.

UV-vis Spectroelectrochemistry: *In situ* spectroelectrochemical analysis was performed in a glass cuvette (path length 1 mm) using a CP|CoFc-MOF working electrode (CP = carbon

paper, AvCarb P50T), a Ag|AgCl|KCl (satd.) reference electrode, and a Pt wire counter electrode. An AvaLight-DHC (deuterium and halogen light source) was used as the light source, and the spectra were recorded using an AvaSpec-2048 spectrometer. The electrode was equilibrated in the electrolyte at the open-circuit potential for 1 hour before applying the oxidative potential. The UV-vis spectrum was recorded at regular intervals.

***Ex situ* HPLC:** HPLC was performed using a Shimadzu Prominence modular setup, comprising of a SIL-20A Auto Sampler, DGU-20A5 Degasser, LC-20AD Liquid Chromatograph, CTO-20A Column Oven and SPD-20A UV-Vis Detector connected with a CBM-20A Communication Bus. The LabSolutions software package was utilised for sample acquisition and post-processing. Chronoamperometry was performed at 10 mA cm^{-2} for 2 hours in 0.1M KOH, using a NF counter electrode and Ag|AgCl|KCl (satd.) reference electrode. 50 µL aliquots of electrolyte were taken at minutes 2, 5, 10, 20, 30, 40, 50, 60 and 120. Samples were prepared for HPLC using the 50 µL aliquot of electrolyte and 950 µL of 1% acetic acid solution in 6:4 acetonitrile:water. Samples were analysed using a 6:4 acetonitrile:water solvent mix, with a flow of 0.2 mL s^{-1}. The run time was 15 minutes, with an oven temperature of 30°C and an analysis wavelength of 254 nm.

***In situ* ICP:** In situ ICP-MS measurements were performed using an Agilent 7900 ICP-MS in combination with a custom electrochemical flow cell with an active surface area of 0.07069 cm^2. Agilent's MassHunter 4.3 was used as the operating software. Samples were dropcast onto 5x5 mm carbon paper squares with a one-sided loading of 0.08 ± 0.05 mg. To apply the electric potentials, a BioLogic VSP-300 potentiostat with a Pt counter electrode and an Ag/AgCl reference electrode was used. A 10 mM KOH electrolyte solution was used (higher electrolyte concentrations would lead to clogging of the ICP-MS). The electrochemical stability tests were performed at constant potentials of 1.4 V and 1.5 V vs. RHE, with flow rates of 8.22 mg s^{-1} and 7.259 mg s^{-1} for total measurement times of 2 h. To quantify Fe and Co, calibration with Merck's ICP multi-element standard solution XVI was performed.

EPR and SQUID measurements: Typical measurement parameters used for EPR: Microwave frequency 9.37GHz, microwave power 2 mW, modulation amplitude 5 G, sweep time 120 s, and sweep range 0-17,000 G. Additional high frequency EPR measurements were attempted using a Bruker ER5106QT (Q Band, 34GHz) and ER5106KT (K Band, 24 GHz) Flexline resonator inside an Oxford Instruments CF935 Cryostat cooled by the same ColdEdge Stinger. The bulk magnetic properties of the powders were studied on a Quantum Design MPMS3 SQUID magnetometer. Magnetic Susceptibility measurements in the range 1.8 K to 300 K along with isothermal magnetisation (at 300 K) between 0 and 7 T. Diamagnetic corrections were applied to account for the response of the gelatin capsule and sample holder.

Optimisation of CoFc-MOF structure: The CoFc-MOF structure was optimised based on a ZnFc-MOF previously reported,[2] changing the Zn atoms with Co. Periodic Density Functional Theory (DFT) calculations were carried out using the CP2K software package.[3] The Gaussian and Plane Waves (GPW) method was utilised, a primary TZVP basis set,[4] corresponding GTH pseudopotentials.[5–7] A cut-off energy of 10.8 keV (800 Ry) and relative cut-off energy of 816 eV (60 Ry) to map the primary Gaussian basis set onto the auxiliary plane-wave basis. This converges unit cell energies to within 10meV. Calculations were performed using the Perdew-Burke-Ernzerhof (PBE) XC functional optimised for solid systems (PBEsol),[8,9] with Grimme's D3 dispersion correction.[10,11] A 2×8×4 Monkhorst-Pack k-point mesh grid was used for all calculations. SCF convergence was set to the largest density matrix element changing by <1.0E-6 and fermi-dirac smearing was used with an effective electronic temperature of 500 K. Geometry optimisations and cell optimisations were carried out unit maximum forces on atoms were <0.02 eVÅ^{-1} and pressure on the cell <10 MPa.

Supplementary Figures

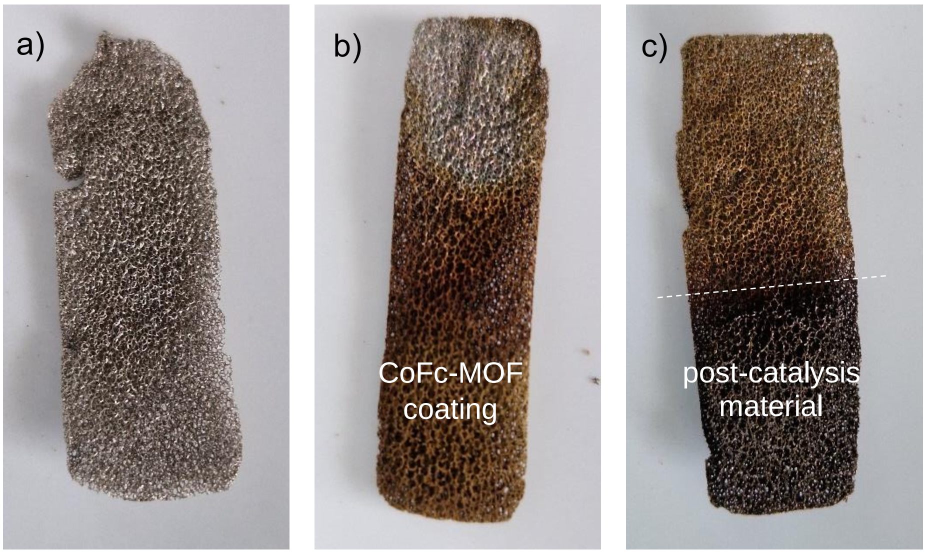

Figure S1. Photographs of (a) blank NF, (b) as synthesised NF|CoFc-MOF, and (c) post-catalysis NF|CoFc-MOF (24 h chronopotentiometry at 10 mA cm^{-2}). In Figure (c), the part of the electrode below the white line was immersed in the electrolyte during chronopotentiometry.

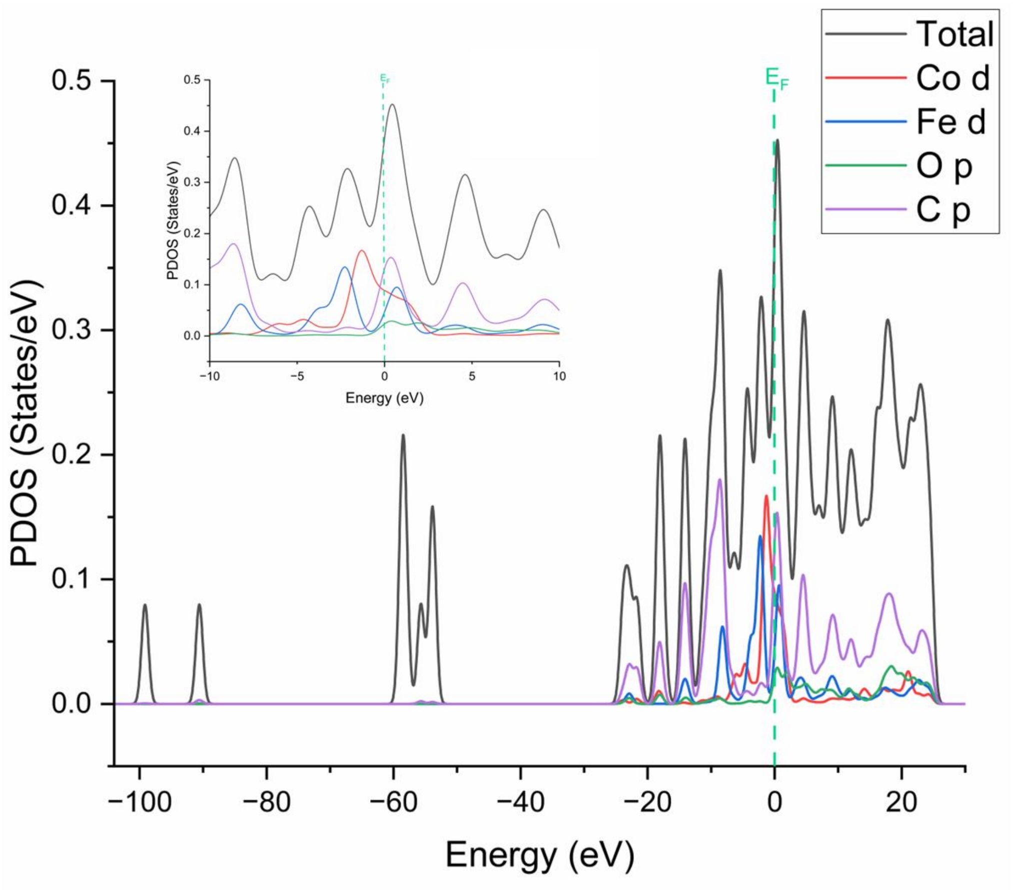

Figure S2. Partial Density of states for the optimised CoFc-MOF. The inset shows a narrow window surrounding the Fermi Energy (E_F).

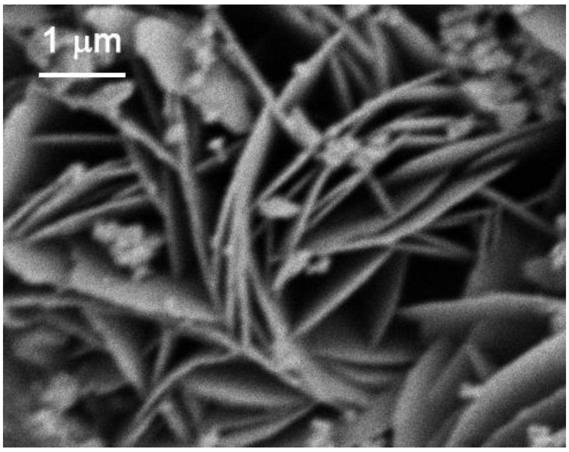

Figure S3. SEM image of bulk CoFc-MOF. The smaller aggregates on the sheets indicate that the bulk material is a mixture of two types of particles with different morphologies.

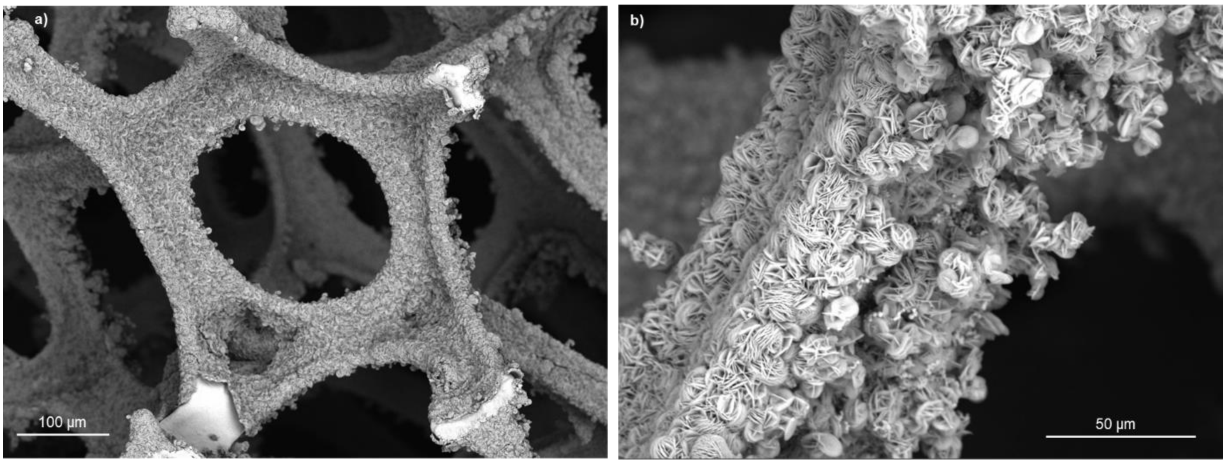

Figure S4. (a and b) SEM image of NF|CoFc-MOF at different magnifications.

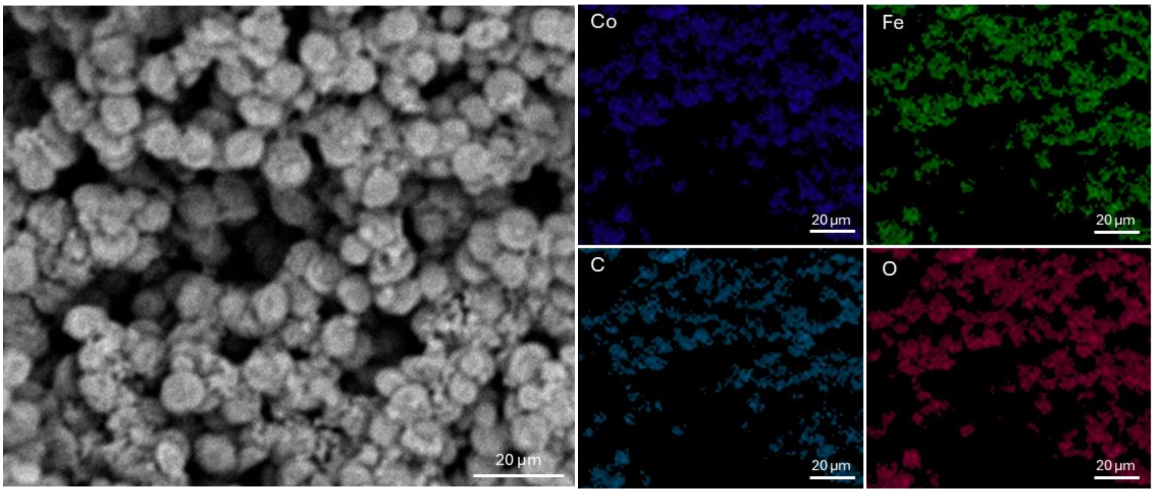

Figure S5. EDX elemental maps of NF|CoFc-MOF

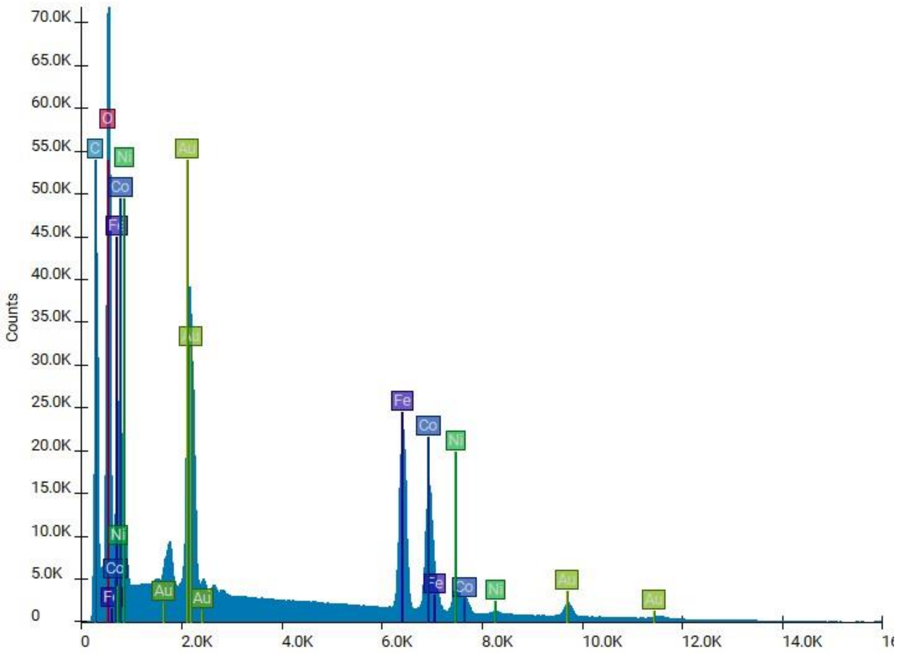

Figure S6. EDS Spectra of NF|CoFc-MOF Samples are coated with 5 nm of gold utilising a sputter coater.

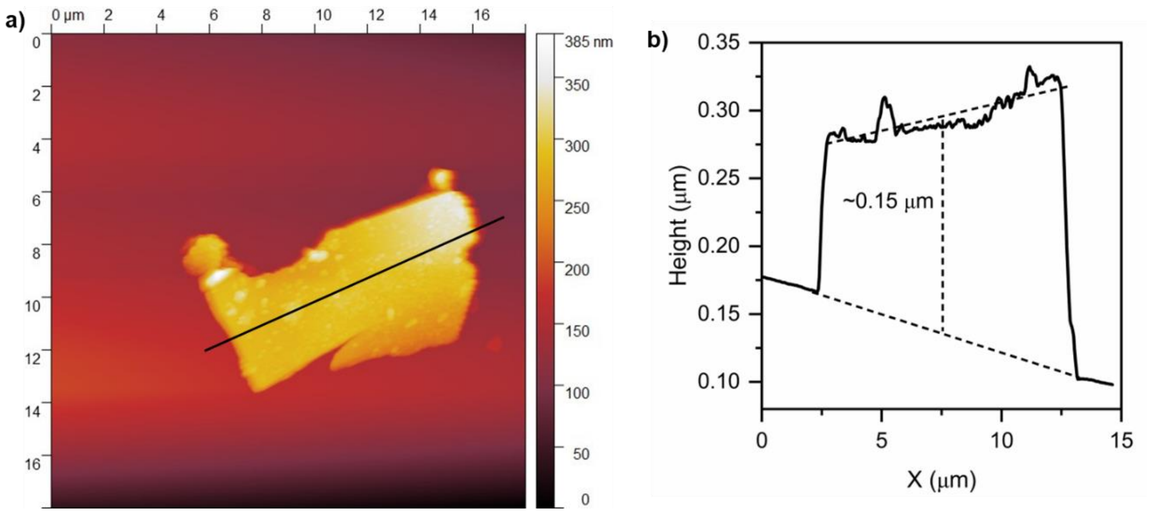

Figure S7. (a) AFM image of a CoFc-MOF flake, and (b) AFM height profile associated with black line.

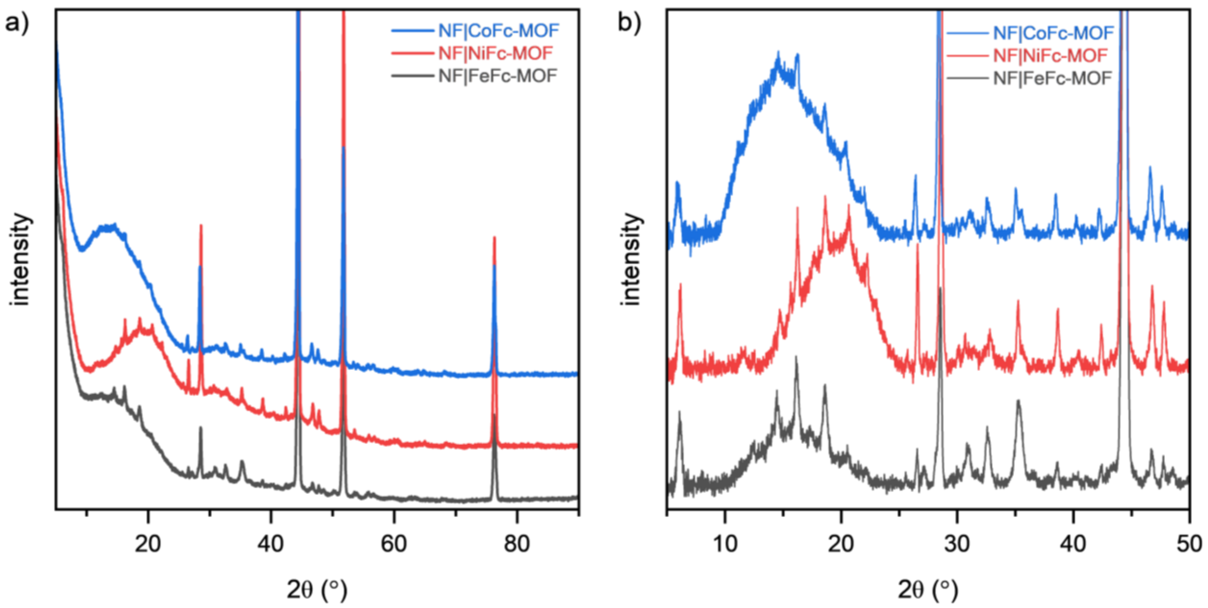

Figure S8. Powder X-ray diffraction patterns of NF|CoFc-MOF, NF|NiFc-MOF, and NF|FeFc-MOF, demonstrating similar structure of the three surface-grown materials. Panel (a) shows the non-baseline corrected data and (b) shows the baseline corrected data in the range 2θ = 5–50°. The intense reflections at ~28°, ~44°, ~52°, and ~76° observed in all three electrodes originate from the sample holder and nickel foam support.

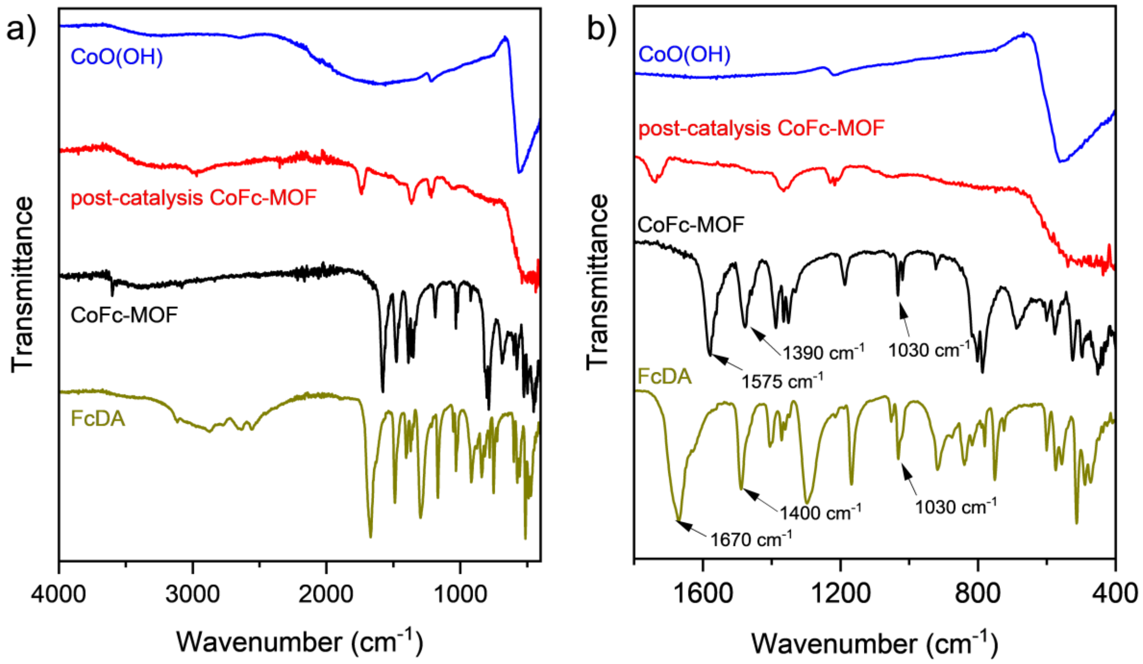

Figure S9. ATR-IR spectra of FcDA, CoFc-MOF, post-catalysis CoFc-MOF (electrode material), and CoOOH. Figure a) shows the full range (400-4000 cm^{-1}) and b) shows the zoom of 400-1700 cm^{-1} region.

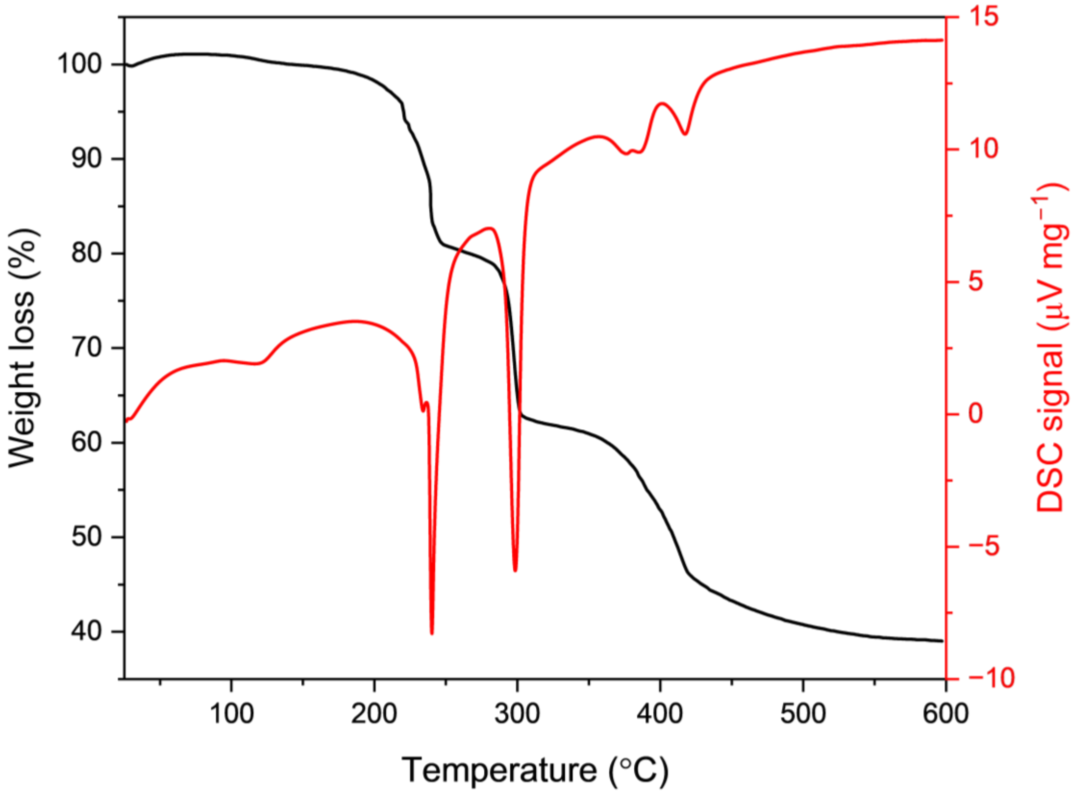

Figure S10. Thermogravimetric analysis (TGA) and differential scanning calorimetry (DSC) of CoFc-MOF recorded at 10 °C min^{-1} heating ramp.

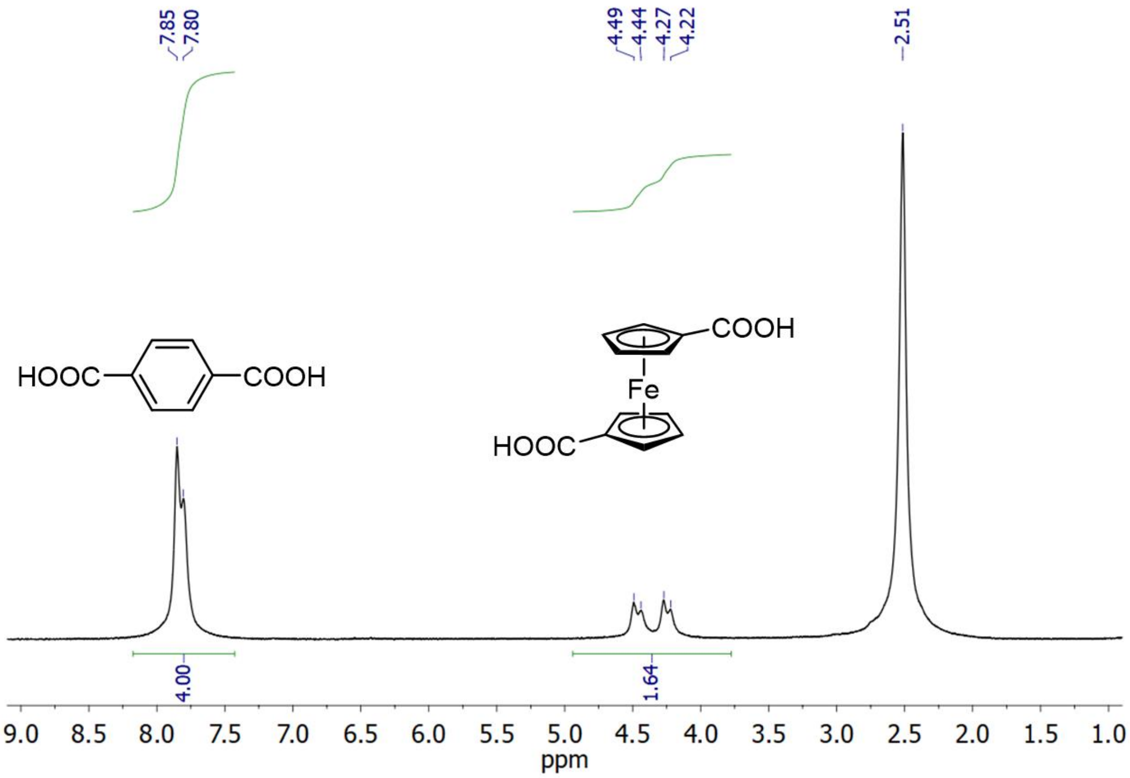

Figure S11. NMR spectrum of CoFc-MOF with terephthalic acid internal reference. 100 µL D_2SO_4 and 500 µL DMSO-d6 were added to 2.5 mg CoFc-MOF. The resulting suspension was heated and sonicated until all particulate was dissolved. 3 mg terephthalic acid (TPA) was added as an internal reference.

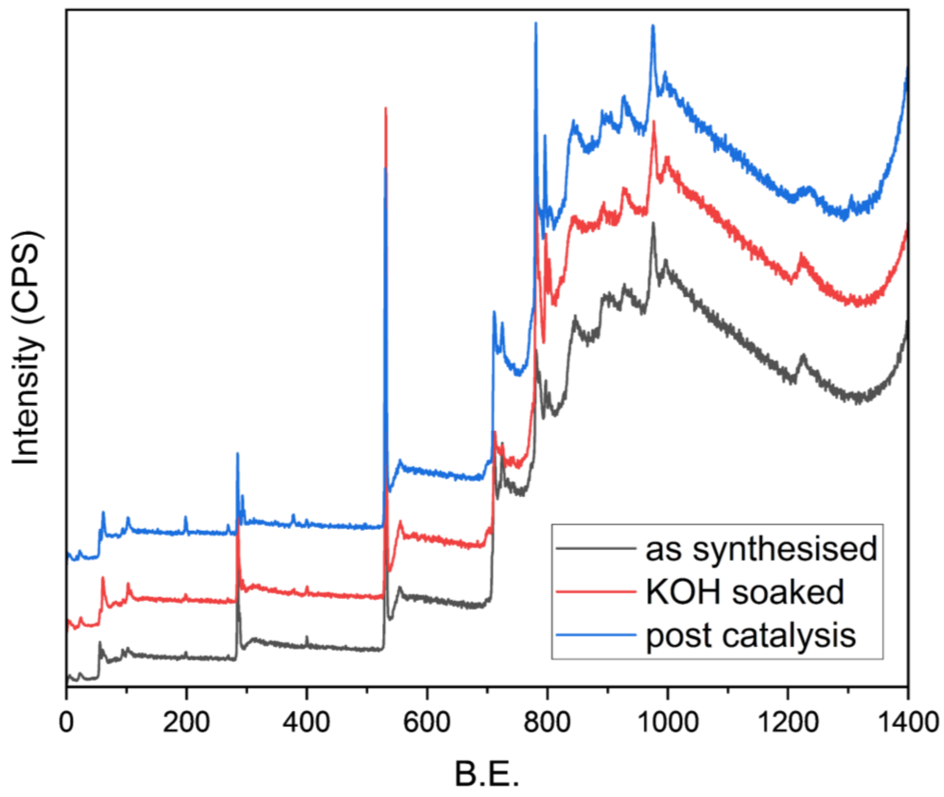

Figure S12. Survey XPS of as-synthesised, KOH-soaked, and post-catalysis CoFc-MOF.

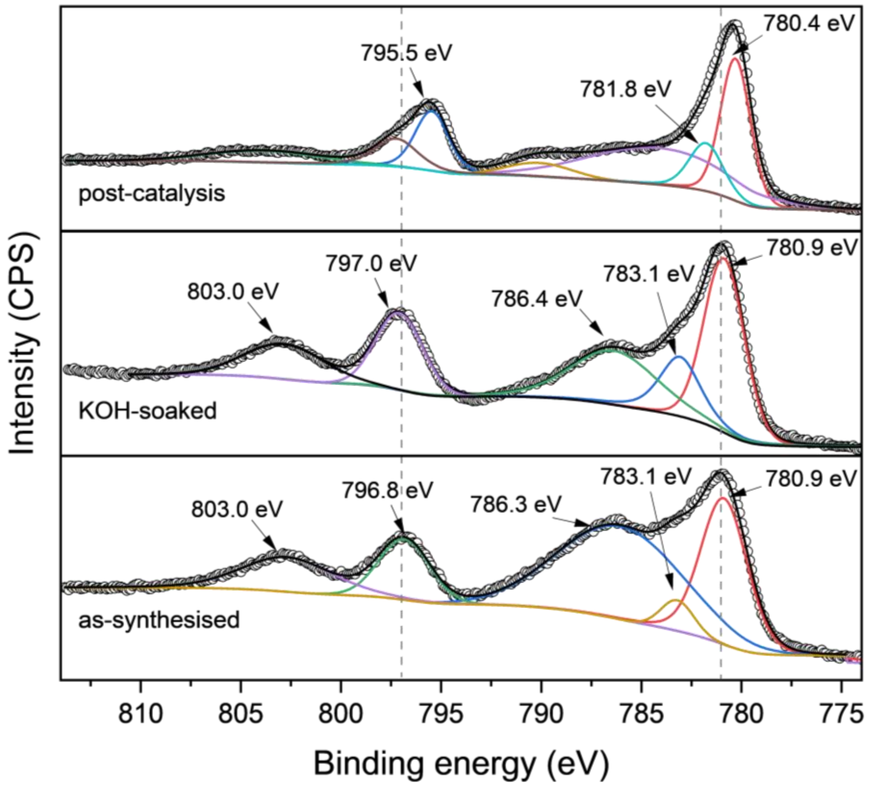

Figure S13. Co 2p XP spectra of as-synthesised, KOH-soaked, and post-catalysis CoFc-MOF. The solid black lines show the envelope of the fitted peaks.

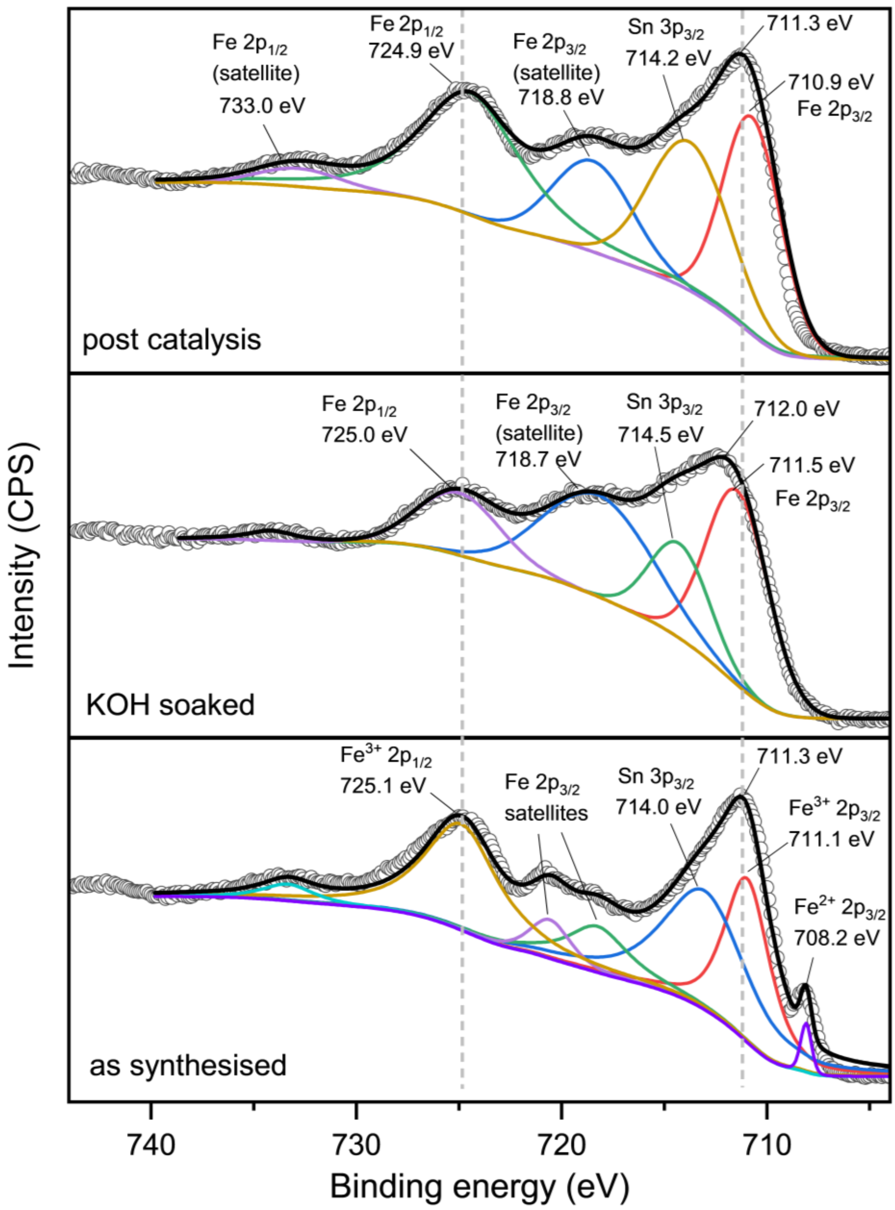

Figure S14. Fe 2p XP spectra of as-synthesised, KOH-soaked, and post-catalysis CoFc-MOF. The solid black lines show the envelope of the fitted peaks.

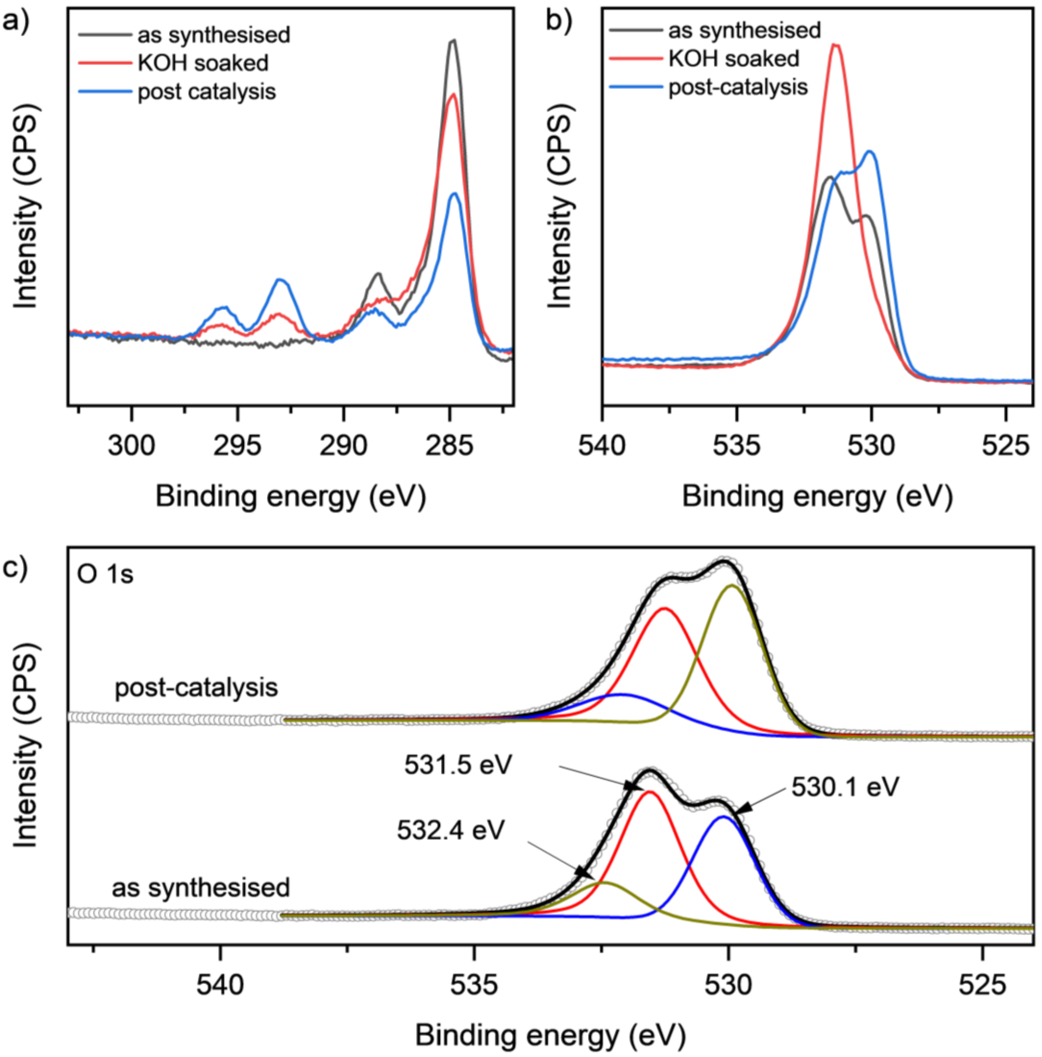

Figure S15. (a) C 1s and (b) O 1s XPS spectra of as-synthesised, KOH-soaked, and post-catalysis CoFc-MOF. The two additional peaks at 292.9 eV and 295.7 eV in the C 1s XPS os KOH-soaked and post-catalysis samples (panel a) correspond to K $2p_{3/2}$ and $2p_{1/2}$ signals, respectively. (c) O 1s XPS peak fit for as-synthesised and post-catalysis samples with the solid black lines showing the envelope of the fitted peaks.

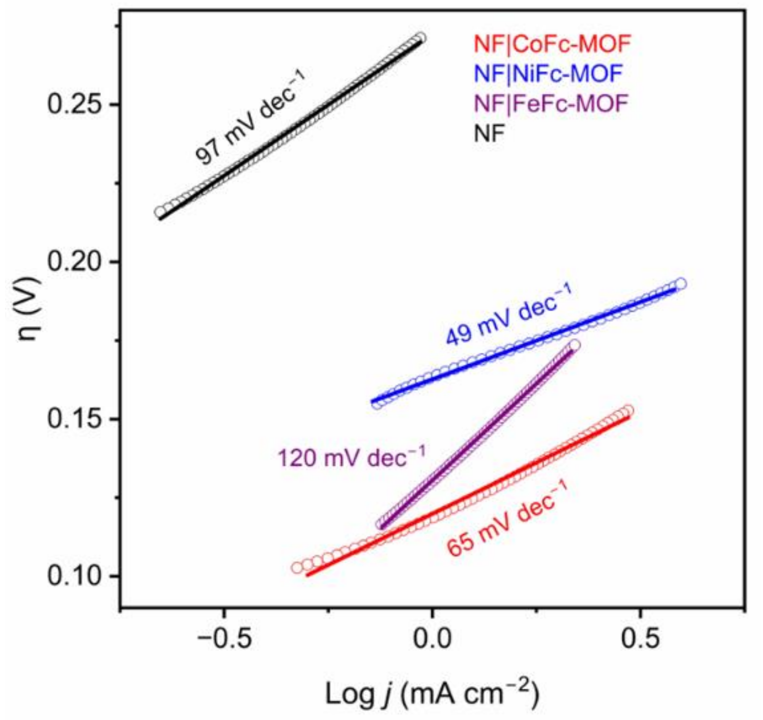

Figure S16. Tafel plot of FcDA-based MOFs derived from LSVs recorded at 5 mV s^{-1}.

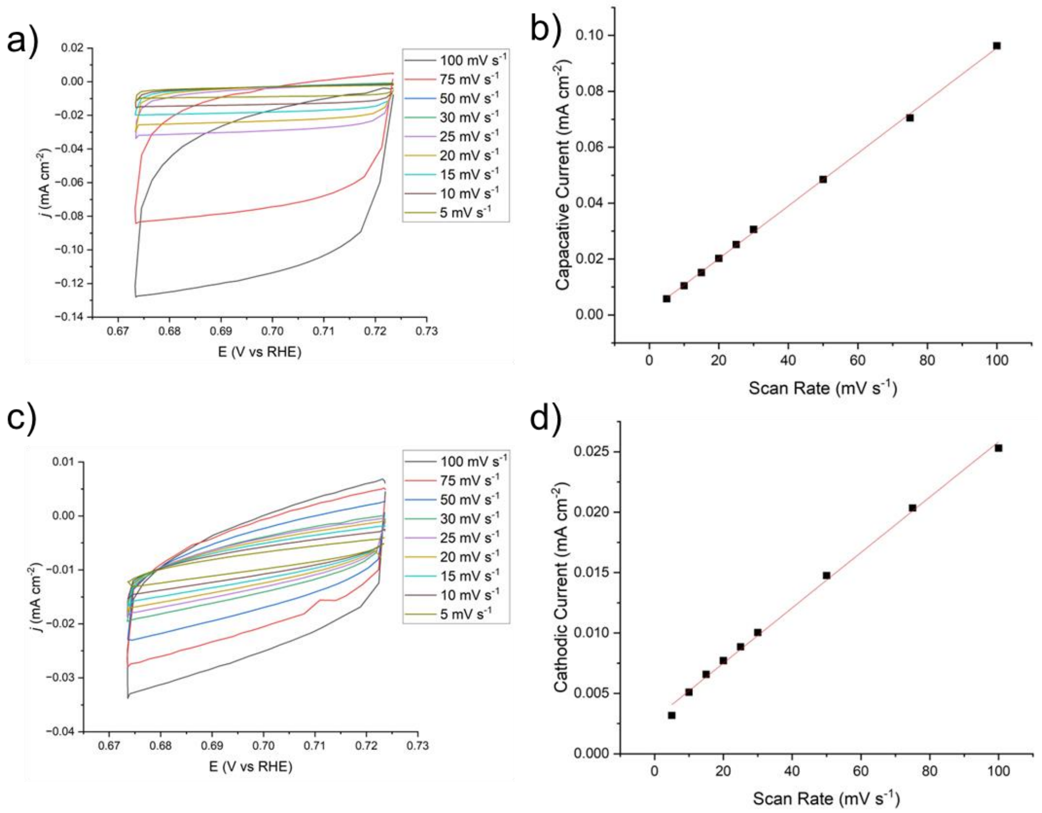

Figure S17. a) CV curves of NF|CoFc-MOF at different scan rates b) Plot of capacitive current of NF|CoFc at 0.7 V vs RHE. C_{dl} = 4.71 mF cm^{-2} c) CV curves of NF at different scan rates d) Plot of capacitive current of NF at 0.7 V vs RHE. C_{dl} = 1.15.

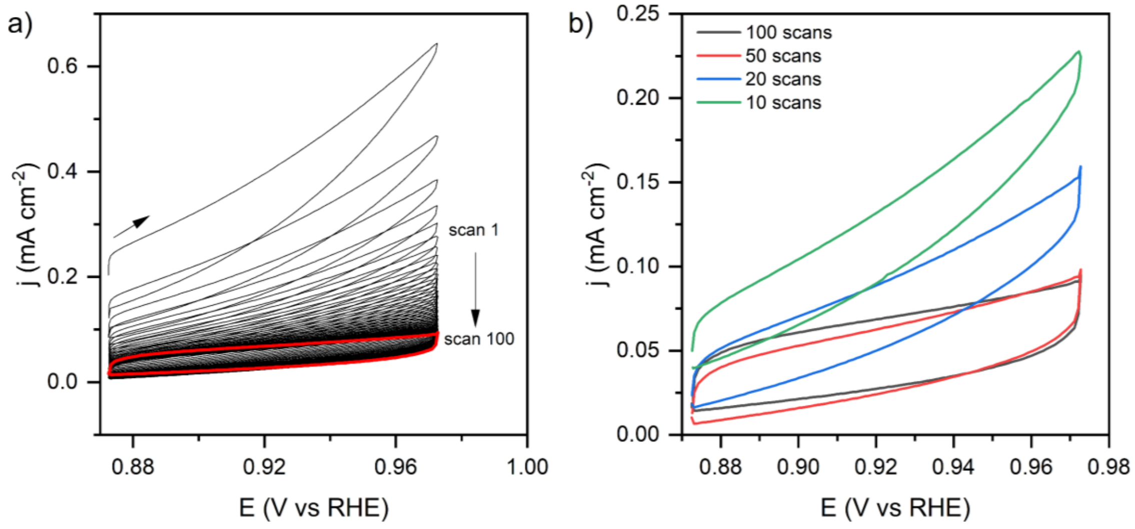

Figure S18. a) Continuous CV of NF|CoFc-MOF in 1M KOH in the non-Faradaic potential window. b) Selected voltammograms showing the change in shape of CV with increasing number of scans. The geometric surface area of the electrode is ~0.5 cm^2.

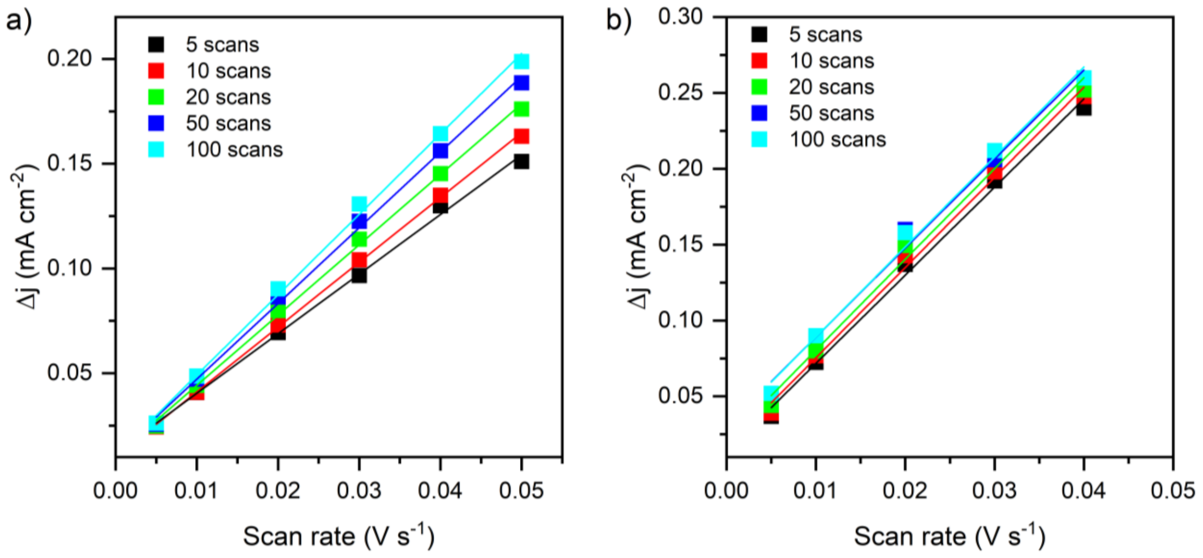

Figure S19. Linear dependence of capacitive current on the scan rate for NF|CoFc-MOF after increasing number of continuous CV scans in the potential window 0.87–0.97 V V (Figure a) and 0.87–1.37 V V (Figure b). The capacitive current densities at varying scan rates were determined from the difference in cathodic and anodic current densities at 0.92 V.

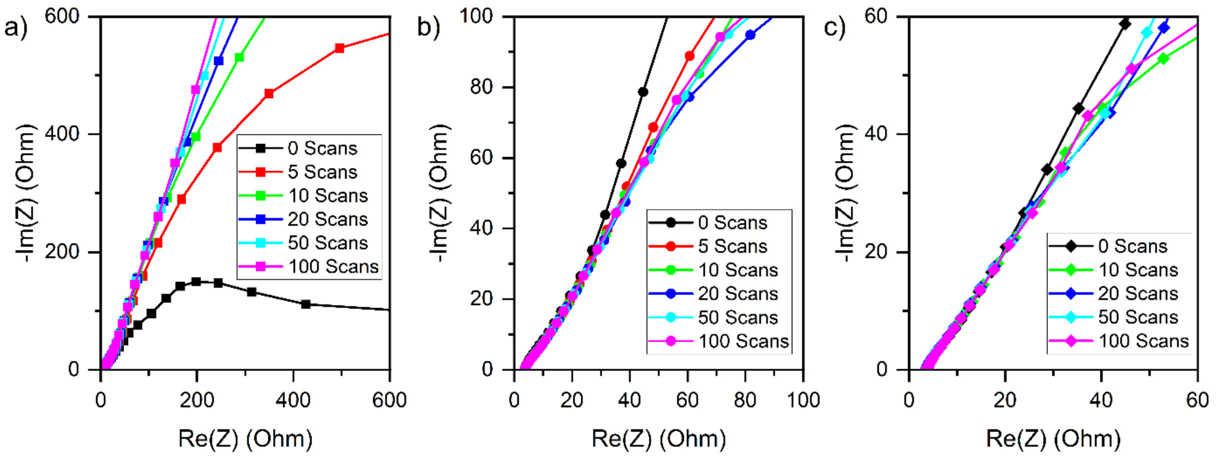

Figure S20. Nyquist plots of NF|CoFc-MOF electrode after CV scans with different cycle numbers. The voltammetric scans were performed over the following potential windows: (a) 0.87–0.97 V, (b) 0.87–1.37 V, and (c) 0.87–1.57 V. The EIS experiments were carried out with an applied potential of 0.87 V vs. RHE in 1.0 M KOH.

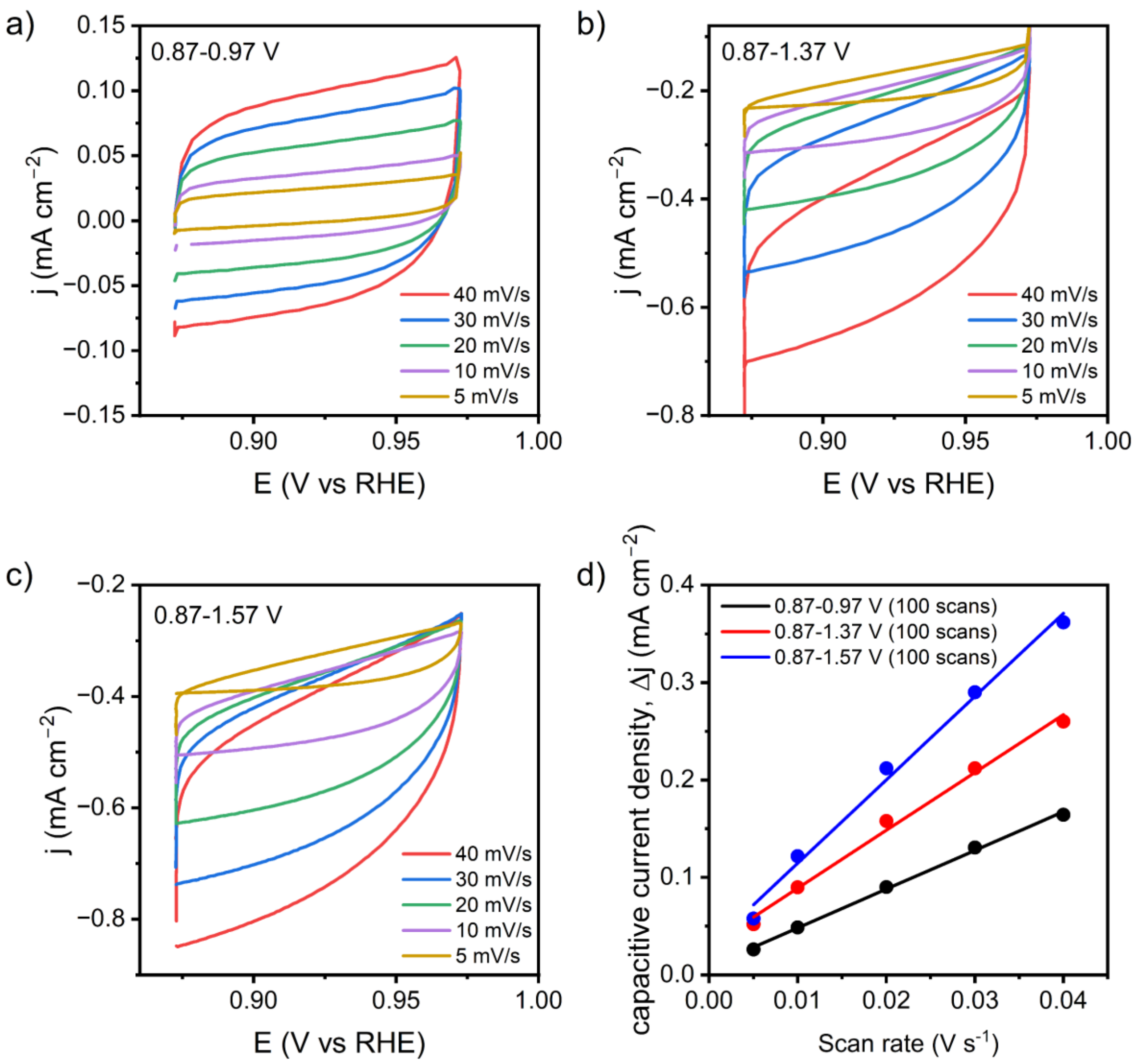

Figure S21. (a-c) CV curves of NF|CoFc-MOF in the non-Faradaic region at different scan rates to compare the double layer capacitance of NF|CoFc-MOF after 100 continuous CV scans over the following potential windows: (a) 0.87–0.97 V, (b) 0.87–1.37 V, and (c) 0.87–1.57 V. (d) Linear dependence of capacitive current density at 0.92 V on the scan rate for NF|CoFc-MOF after 100 CV scans in the potential windows 0.87–0.97 V (black), 0.87–1.37 V (red), and 0.87–1.57 V (blue). The straight lines show the linear fit of the data recorded at scan rates 0.005, 0.01, 0.02, 0.03, and 0.04 V s^{-1}. The double-layer capacitance (C_{dl}) is equal to half of the slope and is proportional to the ECSA of the electrode. C_{dl} = 4.3 mF cm^{-2} (blue trace), 3.0 mF cm^{-2} (red trace), 2.0 mF cm^{-2} (black trace).

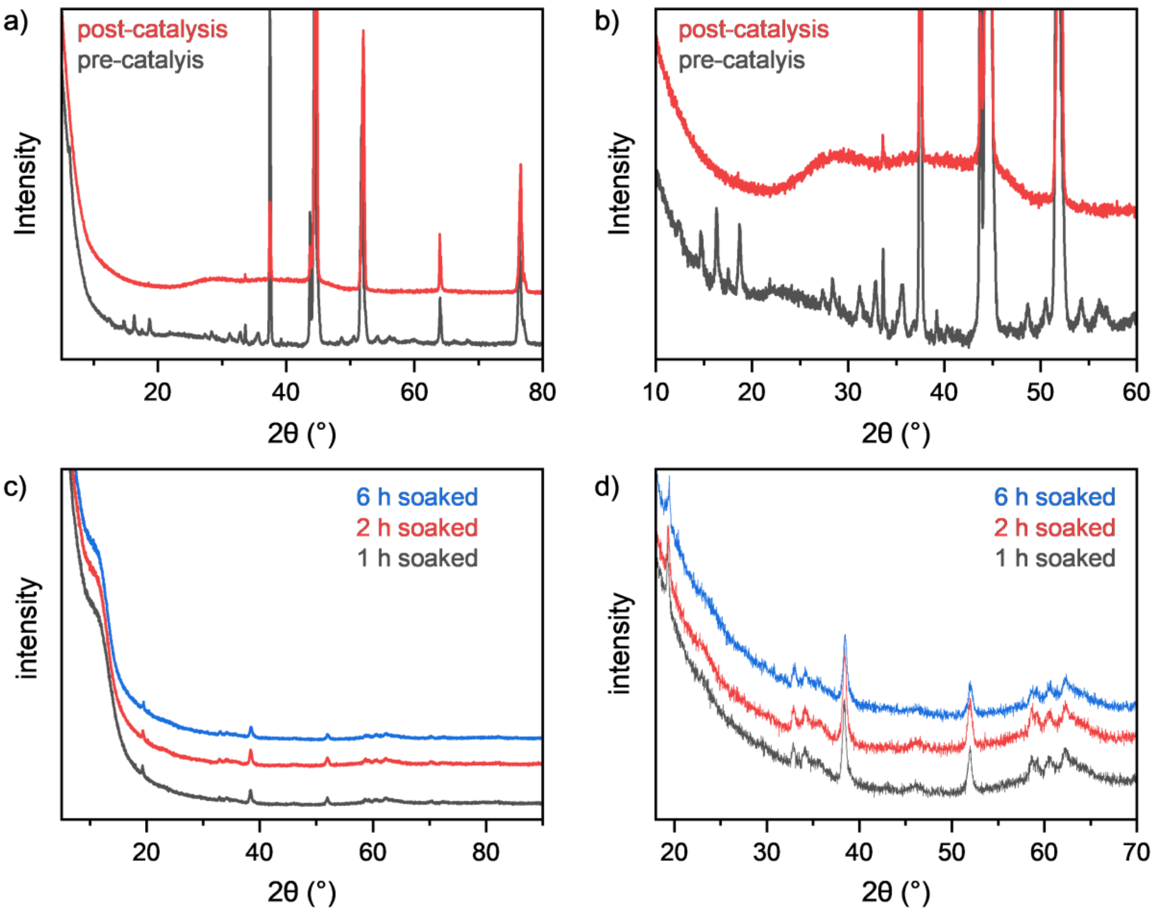

Figure S22. (a, b) PXRD pattern of NF|CoFc-MOF before catalysis and after catalysis (chronopotentiometry at 10 mA cm^{-2} for 6 h). Panel (b) shows zoom of the plot for range 2θ = 10-60°. (c, d) PXRD pattern of CoFc-MOF soaked in 1M KOH for different durations. Panel (d) shows zoom of the plot for range 2θ = 18-90°.

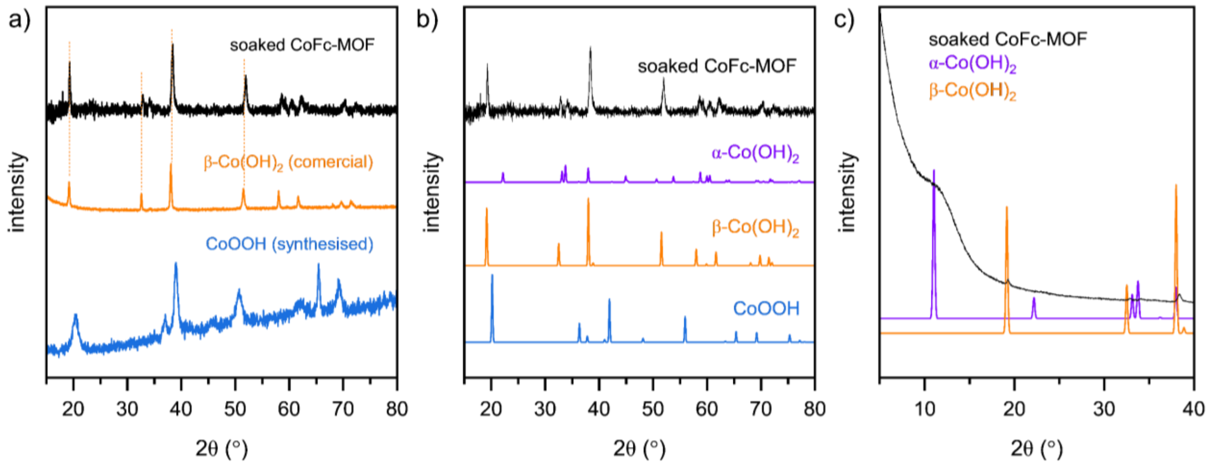

Figure S23. (a) PXRD pattern of KOH-soaked CoFc-MOF compared to that of commercial β-$Co(OH)_2$ and CoOOH (synthesised according to literature protocol); the PXRD data for soaked CoFc-MOF was baseline corrected. (b) PXRD pattern of KOH-soaked MOF is compared against the patterns of β-$Co(OH)_2$ (collection code 257275), α-$Co(OH)_2$ (collection code 172037) and CoOOH (collection code 56288) obtained from the Inorganic Crystal Structure Database. (c) PXRD pattern of KOH-soaked CoFc-MOF (non-baseline corrected) showing the peaks in the smaller 2θ range.

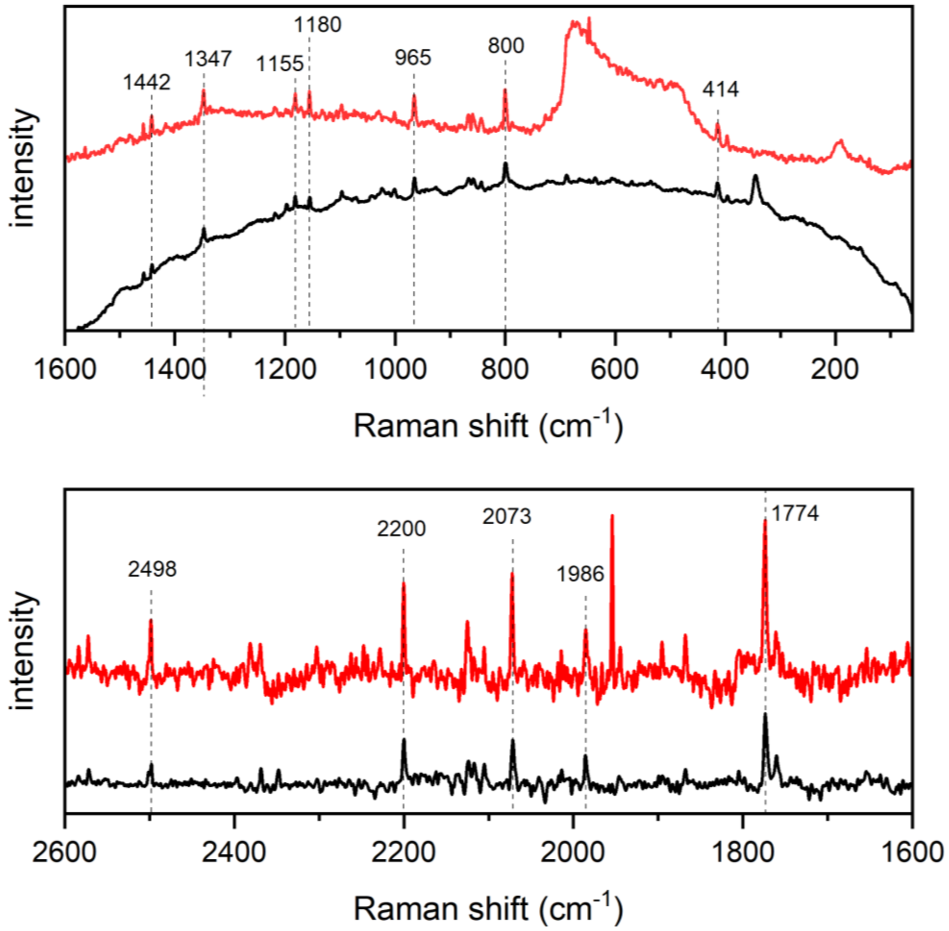

Figure S24. Comparison of the Raman spectra of as-synthesised NF|CoFc-MOF (black trace) and post-catalysis NF|CoFc-MOF (red trace). For the post-catalysis sample, the electrode was used for chronopotentiometry at 10 mA cm-2 for 12 hours. The bottom panel shows 1600–2600 cm^{-1} region and the data were baseline-corrected using the in-built function in OPUS software.

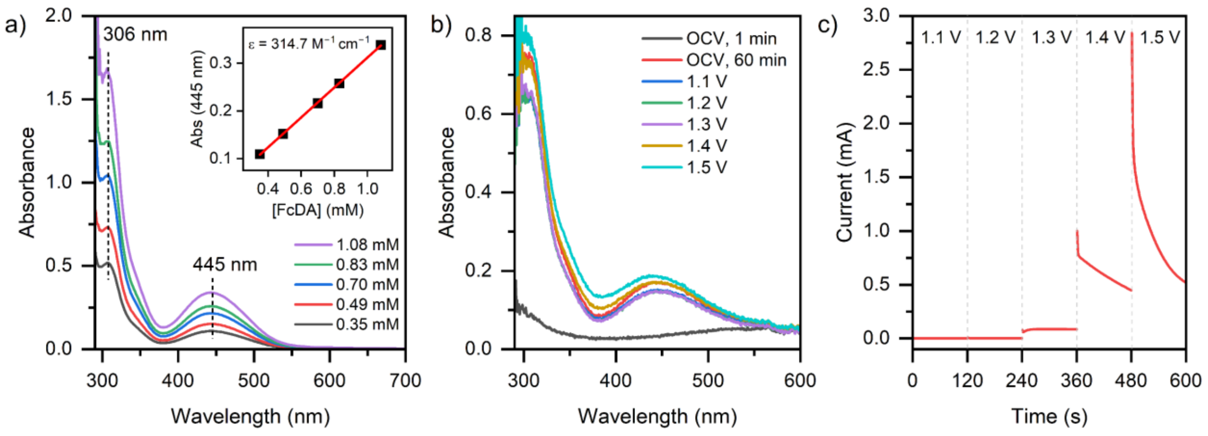

Figure S25. (a) UV-vis spectra of 1,1'-ferrocene dicarboxylic acid (FcDA) in aqueous KOH (1 M) at different concentration. The inset shows the plot of absorbance at 445 nm versus concentration of FcDA which produced a molar extinction coefficient (ε_{445nm}) of 314.7 M^{-1} cm^{-1}. The 306 nm and 445 nm bands are attributed to charge-transfer and d-d transitions, respectively.[12] (b) In situ UV-vis spectroelectrochemistry of the electrolyte during chronopotentiometry with CoFc-MOF electrode at different applied potential (vs. RHE). The catalyst loading on the carbon paper electrode was ~0.8 mg cm^{-2}. The electrode was held at each potential for 2 minutes with stirring before recording the spectrum. The black and red trace show the pre-electrolysis UV-vis spectra (at open circuit voltage, OCV) of the electrolyte after equilibriating the electrode for 1 min and 60 min, respectively. (c) The current vs. time trace for the spectroelectrochemistry experiment showing the anodic current at different applied potential.

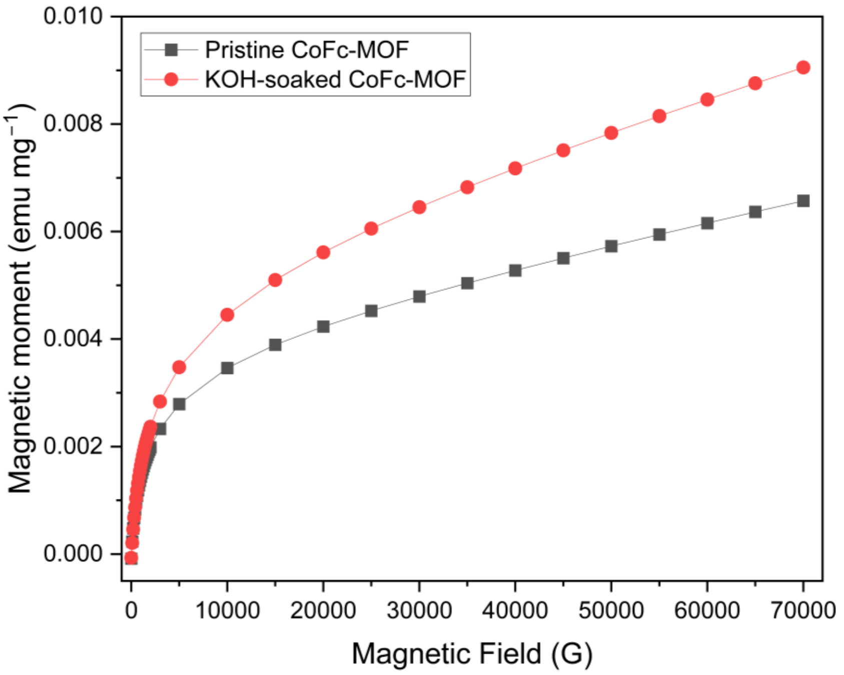

Figure S26. Variable-field magnetization data for pristine and KOH-soaked CoFc-MOF collected at 300 K. The KOH-soaked sample was prepared by soaking the MOF in 1 M KOH for 2 h.

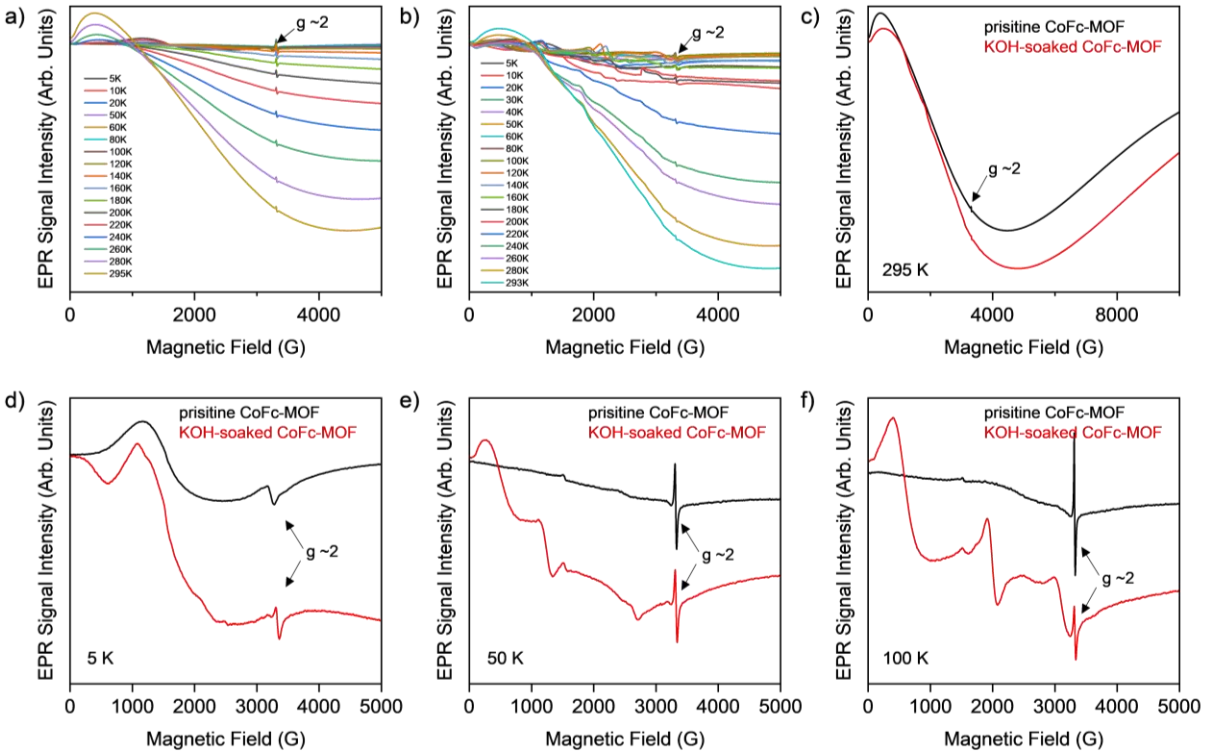

Figure S27. (a, b) Temperature dependent X-band EPR spectra for (a) pristine and (b) KOH-soaked CoFc-MOF. (c-f) Comparison of the X-band EPR spectra of pristine CoFc-MOF (black trace) and KOH-soaked CoFc-MOF (red trace) at (c) 295 K, (d) 5K, (e) 50 K, and (f) 100 K.

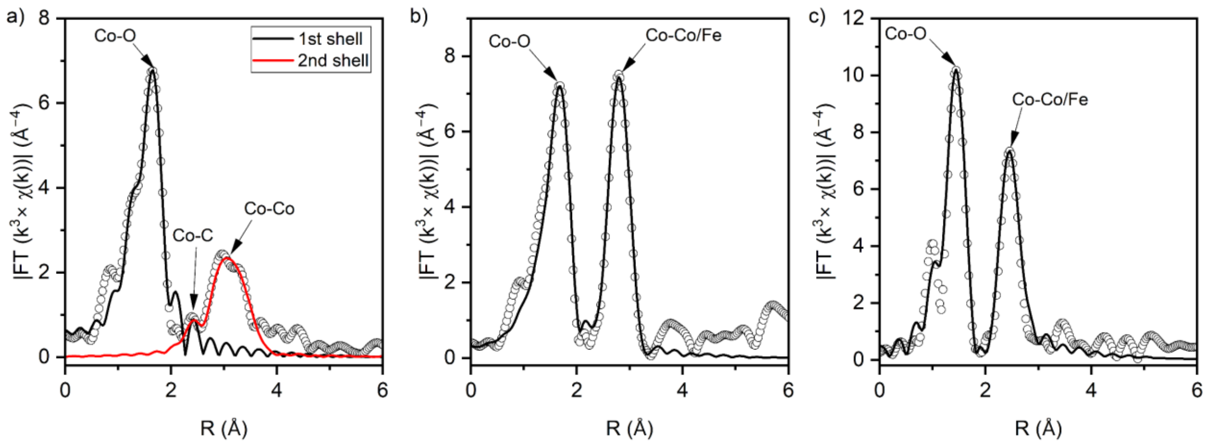

Figure S28. The Fourier transform of e k^3-weighted EXAFS spectra of Co K-edge for (a) pristine CoFc-MOF, (b) KOH-treated CoFc-MOF, and (c) post-catalysis CoFc-MOF. The open circles show the experimental data and the solid lines present the simulations. The fit parameters for the simulations are given in Table S3.

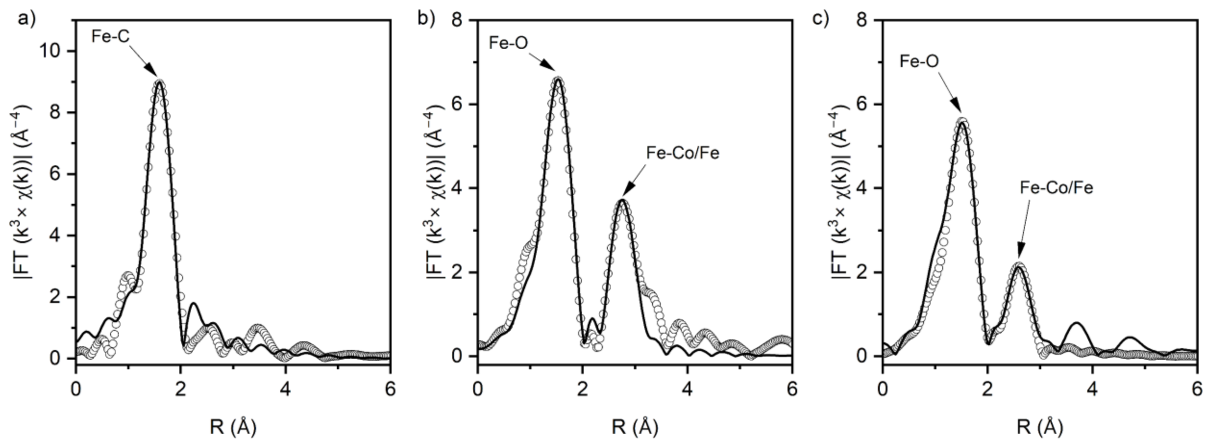

Figure S29. The Fourier transform of e k^3-weighted EXAFS spectra of Fe K-edge for (a) pristine CoFc-MOF, (b) KOH-treated CoFc-MOF, and (c) post-catalysis CoFc-MOF. The open circles show the experimental data and the solid lines present the simulations. The fit parameters for the simulations are given in Table S4.

Table S1. Experimental mass loss of CoFc-MOF observed in the TGA curve, and the calculated mass loss based on a molecular formula of [Co(OH)(FcDA)•DMF].

TG	Measured (%)	Calculated (%)	Event
20-230 °C	19	17.4	Solvent loss (DMF)
230-300 °C	19	43.9	Decarboxylation of FcDA linker
300-400 °C	22		Decomposition of ferrocene
Residual weight	40	38.7	Co_2O_3 + Fe_2O_3

Table S2. Atomic and weight percentages from EDS for pre- and post-catalysis NF|CoFc

Sample	Element	Atomic %	Weight %
Pristine CoFc-MOF	C	62.57	36.81
	O	20.61	16.16
	Fe	9.06	24.71
	Co	5.64	16.22
	Ni	2.13	6.10
Post-catalysis CoFc-MOF	C	31.67	14.49
	O	39.82	24.09
	Fe	12.47	25.89
	Co	8.43	18.63
	Ni	7.62	16.90

Table S3. EXAFS fitting parameters for k^3-weighted Co K-edge data. FEFF input was built using crystal structure data for β-$Co(OH)_2$ (COD # 9009101) and CoOOH (ICSD # 56288).

Sample	path	Coordination number	Bond distance (Å)	σ^2 ($\times10^{-3}$ Å^2)	ΔE (eV)	R factor
CoOOH	Co-O	6	1.90	3.4	–4.1	0.01
	Co-Co	6	2.85	4.2		
$Co(OH)_2$	Co-O	6	2.10	8.2	0.09	0.004
	Co-Co	6	3.18	8.4		
Pristine CoFc-MOF	Co-O	4	2.05	2.2	–7.7	0.03
	Co-O	1.9	1.92	3.6		
	Co-C	1.9	2.70	7.8		
	Co-Co	1.8	3.26	6.0		
	Co-Co	1.7	3.65	8.1		
	Co-Co	1.7	3.41	8.0		
Soaked CoFc-MOF	Co-O	5.6	2.09	7.6	1.18	0.01
	Co-Co/Fe	5.8	3.15	9.7		
Post-catalysis CoFc-MOF	Co-O	3.9	1.87	2.2	–6	0.03
	Co-O	1.4	1.97	2.5		
	Co-Co/Fe	3.3	2.84	1.9		
	Co-Co/Fe	1.9	2.79	2		

Table S4. EXAFS fitting parameters for k^3-weighted Fe K-edge data. FEFF input was built using crystal structure data for ferrocene and Fe(III) layered double hydroxide (ICSD # 159700).

sample	path	Coordination number	Bond distance (Å)	σ^2 ($\times 10^{-3}$ Å^2)	ΔE (eV)	R factor
Pristine CoFc-MOF	Fe-C	10	2.07	7.2	5.68	0.04
Soaked CoFc-MOF	Fe-O	5.8	2.01	9.8	–3.22	0.02
	Fe-Fe/Co	4.1	3.14	9.8		
Post-catalysis CoFc-MOF	Fe-O	5.4	2.00	8.6	–2.50	0.03
	Fe-Fe/Co	3.3	2.97	15.1		

References

(1) Yang, J.; Liu, H.; Martens, W. N.; Frost, R. L. Synthesis and Characterization of Cobalt Hydroxide, Cobalt Oxyhydroxide, and Cobalt Oxide Nanodiscs. *The Journal of Physical Chemistry C* **2010**, *114* (1), 111–119. https://doi.org/10.1021/jp908548f.

(2) Dong, G.; Hong, M.; Chun-ying, D.; Feng, L.; Qing-jin, M. Novel Ferrocene-Based Mixed-Metal Coordination Polymers. *Journal of the Chemical Society, Dalton Transactions* **2002**, *13* (13), 2593–2594. https://doi.org/10.1039/b201604b.

(3) Kühne, T. D.; Iannuzzi, M.; Del Ben, M.; Rybkin, V. V.; Seewald, P.; Stein, F.; Laino, T.; Khaliullin, R. Z.; Schütt, O.; Schiffmann, F.; Golze, D.; Wilhelm, J.; Chulkov, S.; Bani-Hashemian, M. H.; Weber, V.; Borštnik, U.; Taillefumier, M.; Jakobovits, A. S.; Lazzaro, A.; Pabst, H.; Müller, T.; Schade, R.; Guidon, M.; Andermatt, S.; Holmberg, N.; Schenter, G. K.; Hehn, A.; Bussy, A.; Belleflamme, F.; Tabacchi, G.; Glöß, A.; Lass, M.; Bethune, I.; Mundy, C. J.; Plessl, C.; Watkins, M.; VandeVondele, J.; Krack, M.; Hutter, J. CP2K: An Electronic Structure and Molecular Dynamics Software Package - Quickstep: Efficient and Accurate Electronic Structure Calculations. *J Chem Phys* **2020**, *152* (19), 194103. https://doi.org/10.1063/5.0007045.

(4) VandeVondele, J.; Hutter, J. Gaussian Basis Sets for Accurate Calculations on Molecular Systems in Gas and Condensed Phases. *J Chem Phys* **2007**, *127* (11). https://doi.org/10.1063/1.2770708.

(5) Goedecker, S.; Teter, M.; Hutter, J. Separable Dual-Space Gaussian Pseudopotentials. *Phys Rev B* **1996**, *54* (3), 1703–1710. https://doi.org/10.1103/PhysRevB.54.1703.

(6) Hartwigsen, C.; Goedecker, S.; Hutter, J. Relativistic Separable Dual-Space Gaussian Pseudopotentials from H to Rn. *Phys Rev B* **1998**, *58* (7), 3641–3662. https://doi.org/10.1103/PhysRevB.58.3641.

(7) Krack, M. Pseudopotentials for H to Kr Optimized for Gradient-Corrected Exchange-Correlation Functionals. *Theor Chem Acc* **2005**, *114* (1–3), 145–152. https://doi.org/10.1007/s00214-005-0655-y.

(8) Perdew, J. P.; Burke, K.; Ernzerhof, M. Generalized Gradient Approximation Made Simple. *Phys Rev Lett* **1996**, *77* (18), 3865–3868. https://doi.org/10.1103/PhysRevLett.77.3865.

(9) Perdew, J. P.; Ruzsinszky, A.; Csonka, G. I.; Vydrov, O. A.; Scuseria, G. E.; Constantin, L. A.; Zhou, X.; Burke, K. Restoring the Density-Gradient Expansion for Exchange in Solids and Surfaces. *Phys Rev Lett* **2008**, *100* (13), 136406. https://doi.org/10.1103/PhysRevLett.100.136406.

(10) Grimme, S.; Antony, J.; Ehrlich, S.; Krieg, H. A Consistent and Accurate *Ab Initio* Parametrization of Density Functional Dispersion Correction (DFT-D) for the 94 Elements H-Pu. *J Chem Phys* **2010**, *132* (15). https://doi.org/10.1063/1.3382344.

(11) Grimme, S. Semiempirical GGA-type Density Functional Constructed with a Long-range Dispersion Correction. *J Comput Chem* **2006**, *27* (15), 1787–1799. https://doi.org/10.1002/jcc.20495.

(12) Gray, H. B.; Sohn, Y. S.; Hendrickson, N. Electronic Structure of Metallocenes. *J Am Chem Soc* **1971**, *93* (15), 3603–3612. https://doi.org/10.1021/ja00744a011.
